# Structures of *Trypanosoma brucei* Methionyl-tRNA Synthetase with Urea-Based Inhibitors Provide Guidance for Drug Design against Sleeping Sickness

**DOI:** 10.1371/journal.pntd.0002775

**Published:** 2014-04-17

**Authors:** Cho Yeow Koh, Jessica E. Kim, Allan B. Wetzel, Will J. de van der Schueren, Sayaka Shibata, Ranae M. Ranade, Jiyun Liu, Zhongsheng Zhang, J. Robert Gillespie, Frederick S. Buckner, Christophe L. M. J. Verlinde, Erkang Fan, Wim G. J. Hol

**Affiliations:** 1 Department of Biochemistry, University of Washington, Seattle, Washington, United States of America; 2 Department of Chemistry, University of Washington, Seattle, Washington, United States of America; 3 Department of Medicine, University of Washington, Seattle, Washington, United States of America; Northeastern University, United States of America

## Abstract

Methionyl-tRNA synthetase of *Trypanosoma brucei* (*Tb*MetRS) is an important target in the development of new antitrypanosomal drugs. The enzyme is essential, highly flexible and displaying a large degree of changes in protein domains and binding pockets in the presence of substrate, product and inhibitors. Targeting this protein will benefit from a profound understanding of how its structure adapts to ligand binding. A series of urea-based inhibitors (UBIs) has been developed with IC_50_ values as low as 19 nM against the enzyme. The UBIs were shown to be orally available and permeable through the blood-brain barrier, and are therefore candidates for development of drugs for the treatment of late stage human African trypanosomiasis. Here, we expand the structural diversity of inhibitors from the previously reported collection and tested for their inhibitory effect on *Tb*MetRS and on the growth of *T. brucei* cells. The binding modes and binding pockets of 14 UBIs are revealed by determination of their crystal structures in complex with *Tb*MetRS at resolutions between 2.2 Å to 2.9 Å. The structures show binding of the UBIs through conformational selection, including occupancy of the enlarged methionine pocket and the auxiliary pocket. General principles underlying the affinity of UBIs for *Tb*MetRS are derived from these structures, in particular the optimum way to fill the two binding pockets. The conserved auxiliary pocket might play a role in binding tRNA. In addition, a crystal structure of a ternary *Tb*MetRS•inhibitor•AMPPCP complex indicates that the UBIs are not competing with ATP for binding, instead are interacting with ATP through hydrogen bond. This suggests a possibility that a general ‘ATP-engaging’ binding mode can be utilized for the design and development of inhibitors targeting tRNA synthetases of other disease-causing pathogen.

## Author Summary

Infection by the protozoan parasite *Trypanosoma brucei* causes sleeping sickness, also called human African trypanosomiasis. Without treatment, the disease is fatal yet current therapeutic options are inadequate and better medicines are needed. We have previously reported several potent inhibitors of *T. brucei* methionyl-tRNA synthetase, an essential enzyme involved in the protein biosynthesis. Recently, a new series of the inhibitors was synthesized which has improved membrane permeability over the earlier inhibitors. When applied to mouse with *T. brucei* infection, the new compounds are orally available and reach the central nervous system to reduce parasite loads, and therefore are promising molecules to be developed into antitrypanosomal drug. Here, more inhibitors from this series are reported and tested for their activities. High resolution crystal structures were determined that revealed how these inhibitors bind to the target enzyme. The binding pockets of these inhibitors are thoroughly explored, providing profound insights which are beneficial for further development of MetRS inhibitors against sleeping sickness. A ternary complex of the enzyme, an inhibitor, and an ATP analogue was also determined, indicates that the inhibitor does not compete with ATP for binding. Based on this, a general approach to use inhibitors that engage ATP for binding to tRNA synthetases is proposed.

## Introduction

Human African trypanosomiasis (HAT), also called sleeping sickness, is a disease caused by the protozoan parasite *Trypanosoma brucei*. Up to 60 million people in sub-Saharan Africa are estimated to be at risk for the infection [1]. The disease usually occurs in two stages. In the first, haemolymphatic, stage, the parasites multiply in blood and lymph. In the second, meningoencephalitic, stage, the parasites cross the blood-brain barrier (BBB) to invade the central nervous system. Most of the reported cases of HAT are caused by *T. brucei gambiense* which progresses slowly in months to years. In contrast, HAT caused by *T. brucei rhodesiense* progresses very rapidly in weeks [2]. HAT is uniformly fatal if left untreated. However, currently available treatment options for HAT are largely inadequate mainly due to drug toxicity. All treatment regimens require parenterally administered drugs and only two (melarsoprol and eflornithine) cross the BBB for treatment of late stage HAT [2], [3]. Therefore, new oral antitrypanosomal drugs that are affordable, effective and safe are urgently needed. It is crucial that a new drug is orally available for ease of storage and administration, and crosses the BBB for effective treatment of the late stage of the disease.

The aminoacyl-tRNA synthetases (aaRS) are essential enzymes involved in protein synthesis and hence attractive targets for anti-infective drug design [4]–[6]. Generally, aaRS recognize a specific amino acid and charge it to its cognate tRNA through a two-step reaction: (1) recognition of the amino acid and ATP to form an aminoacyl-adenylate intermediate, and (2) recognition of the cognate tRNA to transfer the aminoacyl group to the 3′-terminal adenosine of the tRNA. In addition, various proofreading or editing mechanisms can be involved to increase the fidelity of translation [7].

Based on an analysis of available structural and functional information of the parasite and human tRNA synthetases, methionyl-tRNA synthetase of *T. brucei* (*Tb*MetRS) was selected as a target for antitrypanosomal drug design. Most eukaryotes have at least two genes encoding MetRS enzymes that are respectively targeted to cytoplasm on the one hand, and mitochondria and chloroplasts on the other hand, forming two subfamilies. Mitochondrial MetRS belongs to MetRS1 subfamily, harbors one so-called connective peptide (CP) ‘knuckle’ – although residues capable of coordinating Zn^2+^ may or may not be present. In contrast, cytoplasmic MetRS belongs to MetRS2 subfamily and has two knuckles, with either one or two Zn^2+^ ions bound [8]–[10]. Remarkably, the *T. brucei* genome revealed only one gene encoding for a MetRS and this belongs to the MetRS1 subfamily [11]. The presence of only one MetRS in *T. brucei* enables the inhibition of protein translation in the cytosol as well as in the mitochondrion through the inhibition of a single enzyme, boosting the potential of targeting *Tb*MetRS for drug development.

*Tb*MetRS has been validated as a possible drug target through RNAi experiments [12]. In addition, a series of aminoquinolone-based inhibitors (ABIs) was shown to have potent antitrypanosomal activity *in vitro* and *in vivo* [12]. To guide further development of the inhibitors, well diffracting crystals of *Tb*MetRS, which only grow in the presence of methionine, were soaked in solutions of ABIs and resulted in high resolution views of protein•ABI complexes [13]. Despite being in the crystalline state, the enzyme showed a large degree of flexibility, resulting in substantially different conformational states when bound with substrate, product or inhibitors. This, along with analysis of other available structures of MetRS, led to the conclusion that conformational selection is the main mechanism by which these compounds bind to *Tb*MetRS. Major conformational states of the enzyme are the ligand-free “F-state”, the Met-bound “M-state”, the intermediate product methionyl-adenosine monophosphate (MAMP)-bound “P-state”, and the inhibitor-bound “I-state”. The binding pockets for ABIs are likely to be present in the F-state, but not M-state and P-state, and are stabilized by the ABIs to drive the population of conformations of the enzyme towards the I-state [13].

Unfortunately, despite their potency, the ABIs have poor membrane permeability and are unlikely to cross the BBB for treating late stage HAT, rendering them of limited use as antitrypanosomal drugs [14]. Therefore, another series of *Tb*MetRS inhibitors, with a urea moiety connected to an aryl group, was designed to replace the aminoquinolone moiety [14]. These urea-based inhibitors (UBIs) inhibit the blood stream form of *T. brucei* in culture with an EC_50_ as low as 150 nM while having minimum toxicity to mammalian cells. Representative UBIs, such as **Chem 1433** and **Chem 1356**, appear to have excellent membrane permeability, enter the central nervous system, have reasonable oral bioavailability, and suppressive activity against the parasite in a mouse model [14]. In culture, resistance to both ABIs and UBIs can be induced, but resistance development is slower than eflornithine and pentamidine [15]. However, the binding mode of UBIs is not obvious without determination of crystal structures, due to the conformational flexibility of *Tb*MetRS. To ascertain their binding mode, and to further understand the structural plasticity of *Tb*MetRS, a total of 15 crystal structures of *Tb*MetRS•UBIs complexes were determined. The collection of UBIs explores a larger chemical, structural and functional diversity than the previously reported ABIs, thus providing a more complete picture of the binding mode of the inhibitors and the concomitant conformational changes of *Tb*MetRS when a spectrum of inhibitors is bound. By analyzing the 23 structures of *Tb*MetRS•Met, *Tb*MetRS•MAMP, *Tb*MetRS•ABI and *Tb*MetRS•UBI complexes now available, general principles of inhibitor binding to *Tb*MetRS, a flexible enzyme, are derived. These include the filling of the two subpockets in the enlarged methionine pocket (EMP), the importance of planarity in the auxiliary pocket (AP) binding moiety, and the hydrogen bonds with a completely conserved Asp. In addition, a ternary complex of *Tb*MetRS with an UBI and the ATP analogue β,γ-methyleneadenosine 5′-triphosphate (AMPPCP) was also determined, providing structural evidence that UBIs are not competing with ATP for the inhibition of *Tb*MetRS.

## Methods

### Protein expression and purification

Methods for *Tb*MetRS expression and purification are as previously reported [13]. Full-length and truncated (237–773) *Tb*MetRS was cloned into the AVA0421 vector for expression in *E. coli*. Based on the truncated protein, site directed mutagenesis of surface residues ^452^KKE^454^ to ARA, all remote from the active site, was required to obtain well diffracting crystals, but solely in the presence of methionine.

Protein was purified by a Ni-NTA affinity column followed by overnight cleavage of the N-terminal hexa-histidine tag using N-terminally histidine tagged 3C protease at 4°C. Cleaved protein was purified by a second Ni-NTA step followed by size-exclusion chromatography on a Superdex 75 column (Amersham Pharmacia Biotech) using a buffer containing 25 mM HEPES, 500 mM NaCl, 2 mM DTT, 5% glycerol, 0.025% NaN_3_ and 10 mM L-methionine at pH 7.0. Purified protein retained five residues of the 3C protease cleavage site (GPGSM) at the N-terminus.

### Synthesis of inhibitors

Unless otherwise stated, all chemicals were purchased from commercial suppliers and used without further purification. The final purity of all compounds was determined by analytical LCMS with Phenomenex Onyx Monolithic C18 column (4.6 mm×100 mm). The products were detected by UV at the detection wavelength of 220 nm. All compounds were determined to be >95% pure by this method. The purification by preparative HPLC was performed on Waters Xterra Prep RP18 OBD 5 µM (19 mm×50 mm) with CH_3_CN/H_2_O and 0.1% TFA as eluent. The mass spectra were recorded with an Agilent Liquid Chromatograph - Ion Trap Mass Spectrometer. NMR spectra were recorded with either a Bruker 500 MHz spectrometer or a Bruker 300 MHz spectrometer at ambient temperature. Synthesis of **Chem 1433**, **Chem 1356**, **Chem 1387**, **Chem 1392**, **Chem 1444** and **Chem 1415** have been previously reported [14]. The synthesis of **Chem 1472**, **Chem 1473**, **Chem 1475**, **Chem 1476**, **Chem 1469**, **Chem 1478**, **Chem 1509** and **Chem 1540** are similar and given in detail in the Supporting Information.

### Thermal shift assay

The thermal shift assay was performed as previously described [12], [14] using 0.5 mg/mL for *Tb*MetRS, 100 µM of inhibitor, and 5% DMSO. The assays were repeated three times independently.

### *T. brucei* methionyl-tRNA synthetase aminoacylation assay

Compound IC_50_s were determined in the *T. brucei* methionyl-tRNA synthetase aminoacylation assay as previously described [12], [14] except 10 U/mL of pyrophosphatase was used per reaction.

### *T. brucei* growth inhibition assay

*T. brucei brucei* (bloodstream form strain 427 from K. Stuart, Seattle BioMed, Seattle, WA) were used for EC_50_ measurements as previously described [12], [14].

### Protein crystallization

The truncated *Tb*MetRS surface mutant was crystallized following procedures reported earlier [13]. Briefly, the crystals were obtained by vapor diffusion using sitting drops equilibrated against a reservoir containing 2.0 to 2.3 M (NH_4_)_2_SO_4_, 0.2 M NaCl and 0.1 M sodium cacodylate pH 6.2 to 6.8. The drops consisted of 1 µL protein at 10 mg/mL plus 1 µL of the reservoir solution and additional 10 mM L-methionine and 1 mM tris(2-carboxyethyl)phosphine. Crystals grew in 1–2 days at room temperature.

### Soaking of inhibitors

To obtain enzyme•inhibitor complexes, *Tb*MetRS•Met crystals were soaked in a cryo-solution containing the inhibitors as previously described [13]. Briefly, crystals were soaked in a 10 µL solution obtained by mixing 1 µL of 20 mM inhibitor in 20% DMSO, 4 µL reservoir solution and 5 µL 60% glycerol (as cryoprotective agent) in protein buffer. Crystals usually disintegrated when soaked longer than several minutes and, typically, required to be flash frozen in liquid nitrogen within one minute.

### Data collection and structure determination

**Figure 1:**
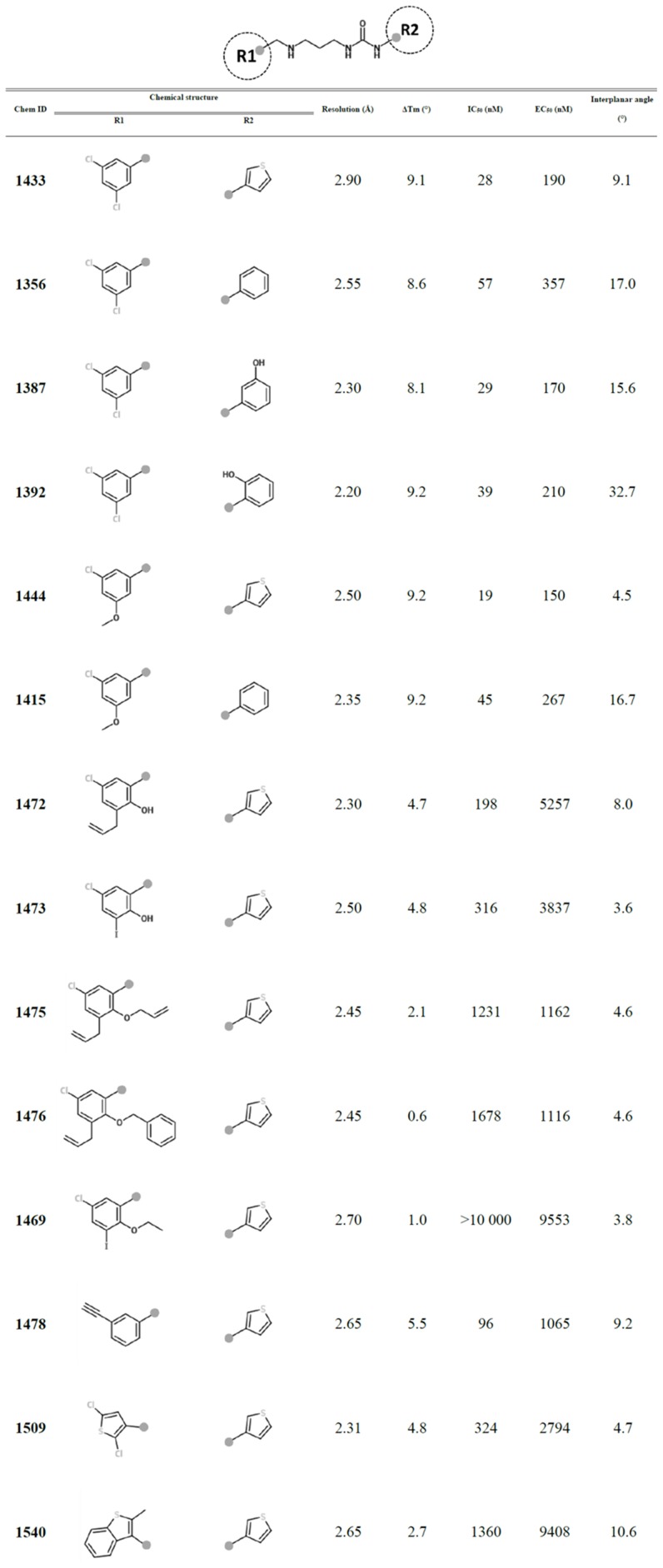
Chemical structures of UBIs and their activities.

All data were collected under cryogenic conditions. For crystals soaked with compounds **Chem 1433**, **Chem 1469** and **Chem 1540**, data were collected in home source facility using a MicroMax-007 HF rotating anode (Rigaku) equipped with VariMax HF (Osmic) monochromator and a Saturn 994 (Rigaku) CCD detector at a wavelength of 1.54 Å. For crystals soaked with compounds **Chem 1356**, **Chem 1472**, **Chem 1473**, **Chem 1475**, **Chem 1476**, **Chem 1478**, **Chem 1509** and with **Chem 1433**•AMPPCP•Mg^2+^, data were collected at Stanford Synchrotron Radiation Lightsource synchrotron beamlines 9-2 and 12-2 at wavelength of 1 Å. For crystals soaked with compounds **Chem 1387**, **Chem 1392**, **Chem 1444** and **Chem 1415**, data were collected at Advanced Light Source synchrotron beamlines 8.2.1 and 8.2.2 at wavelength of 1 Å. All data were processed with HKL2000 (Table S1) [16]. Previously reported structures of *Tb*MetRS [13] were used as search models for phase determination by molecular replacement using the program Phaser [17]. Iterated building/rebuilding and refinement of models, including the use of translational/libration/screw (TLS) groups [18] in refinement, were performed using Coot [19] and REFMAC5 [20], respectively. The refinement restraints for all ligands were generated by the Grade web server [21] and modified based on our survey of crystal structures deposited in the Cambridge Crystallographic Database Center. The structure validation server MolProbity was used throughout the process to monitor the progress of structure determination [22]. All refined structures showed good statistics with no outliers in Ramachandran plots according to MolProbity. The final crystallographic refinement statistics are given in Table S1. The interplanar angles between the urea and the R2 group as listed in Figure 1 were calculated with the program Geomcalc in the CCP4 program suite [23]. Figures were created and rendered with Pymol [24]. Superposition of structures was carried out with Pymol, which first aligns proteins by sequence, followed by structural alignment using five cycles of refinement to improve the fit by discarding pairs with high relative variability.

### Coordinates and structure factors deposition

Coordinates and structure factors for *Tb*MetRS in complex with compound **Chem 1433, Chem 1356, Chem 1387, Chem 1392, Chem 1444, Chem 1415, Chem 1472, Chem 1473, Chem 1475, Chem 1476, Chem 1469, Chem 1478, Chem 1509** and **Chem 1540** are deposited in the Protein Data Bank under PDB ID: 4MVW, 4MVX, 4MVY, 4MW0, 4MW1, 4MW5, 4MW2, 4MW4, 4MWE, 4MW6, 4MW7, 4MW9, 4MWB and 4MWC, respectively. Coordinates and structure factors for *Tb*MetRS in complex with **Chem 1433** and AMPPCP are deposited in the Protein Data Bank under PDB ID 4MWD.

## Results

### Diversity of, and inhibition by, urea-based inhibitors (UBIs)

All 14 UBIs reported here have two aromatic moieties, R1 and R2, connected by a *N*-methylpropylamine linker, as shown on the first line of Figure 1. R1 is typically a substituted phenyl ring, a dichlorothiophene or a benzothiophene moiety. R2 is linked directly to a urea moiety which in turn is connected to the linker. R2 is either a phenyl ring, a hydroxyl-phenyl group, or a thiophene ring (Figure 1). The UBIs were tested for their ability to inhibit growth of *T. brucei* in cell culture (EC_50_) and aminoacylation activity of purified *Tb*MetRS (IC_50_). Changes in melting temperatures (ΔT_m_) of *Tb*MetRS in the presence of the inhibitors were also determined and range from 0.6 to 9.2°C (Figure 1). The UBIs cover a wide range of potencies, with IC_50_ values vary from 19 nM to more than 10,000 nM (Figure 1).

### Crystal structures

Crystal structures of 15 *Tb*MetRS•UBIs complexes were determined with good statistics and geometry, without outliers in Ramachandran plots according to the MolProbity server (Table S1). The structures contain two subunits of *Tb*MetRS per asymmetric unit. The Rossmann fold catalytic core of the enzyme contains an inserted connective peptide (CP) domain which can be further divided into two parts: (1) the ‘CP base’, which is formed by two antiparallel strands spanning from Asp353 to Tyr363 and from Thr398 to Arg408; and (2) the ‘CP knuckle’, formed by residues Ser364 to Val397 between the CP based strands (Figure S1). The single CP knuckle of *Tb*MetRS lacks residues needed for the coordination of Zn^2+^ and hence, although the CP knuckle is well defined in all structures, there is no zinc ion bound.

**Figure 2:**
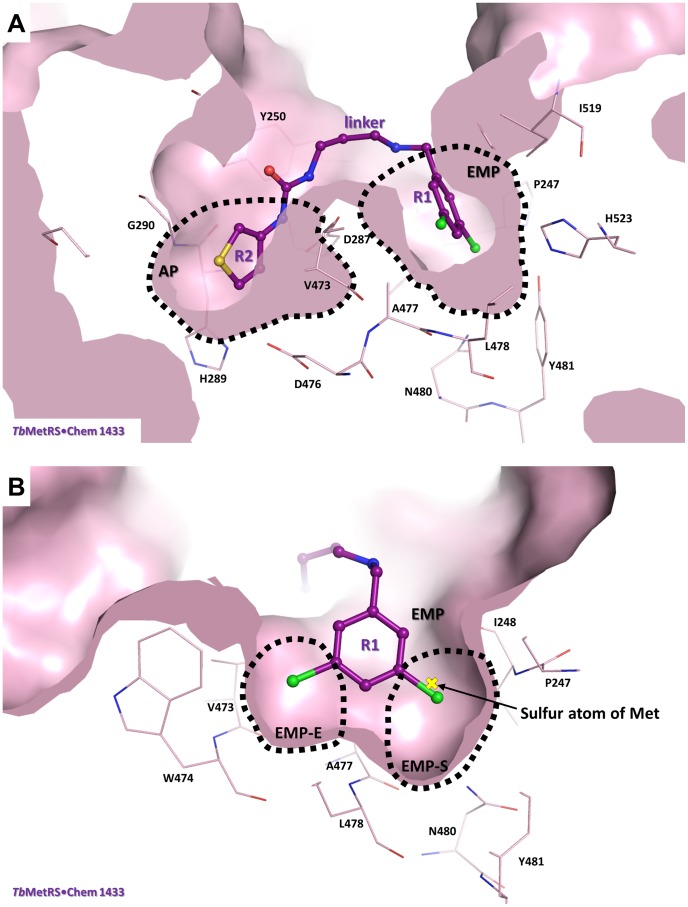
Typical binding mode of an UBI to *Tb*MetRS. (A) The structure of *Tb*MetRS•**Chem 1433** is used as the prototypic complex to depict the binding mode of the UBIs in which the R1 moiety binds to the EMP and urea-R2 moiety binds to the AP. R1 and urea-R2 moieties are connected by the *N*-methylpropanamine linker, which is mostly solvent exposed. **Chem 1433** is shown in ball and stick model in deep purple. *Tb*MetRS is shown in surface representation in light pink with residues within a 4.5 Å radius from **Chem 1433** as stick model in light pink. Also see Figure S3 for interactions between **Chem 1433** and *Tb*MetRS in the EMP and the AP. (B) Binding of inhibitor is accompanied by movement of multiple residues in the active site compared to the Met-bound M-state (PDB code 4EG1). In the EMP, two subpockets, the EMP-S and the EMP-E, can be discerned. Both subpockets are lined mostly by hydrophobic residues, some shown in stick model in light pink. Superposition of *Tb*MetRS•Met complex (not shown) onto *Tb*MetRS•**Chem 1433** (light pink) showed that Met occupies the EMP-S with the sulfur atom (marked as yellow cross) occupying essentially the same position as one of the *meta*-Cl in **Chem 1433**. Val473, Trp474 and Phe522 moved significantly to form the EMP-E. Also see Figure S3A for further details of the interactions within the EMP.

Soaking of UBIs into the *Tb*MetRS•Met crystals results in drastically different responses of the two subunits in the asymmetric unit: Met is retained in subunit A but inhibitor is bound in subunit B (Figure S2). Binding of inhibitors is accompanied by extensive conformational changes in subunit B [13]. Movement of multiple residues near the active site enlarges the initially small Met pocket, forming an enlarged methionine pocket (EMP). In addition, a new, previously unobserved, auxiliary pocket (AP) is formed next to the EMP. UBIs bind to both pockets, with R1 occupying the EMP and urea-R2 filling the AP (Figure 2A). The linker is crucial for properly connecting R1 and R2 such that these moieties can be optimally inserted into the EMP and the nearby AP, but is itself mostly solvent exposed. **Chem 1433** will be discussed as the prototypic UBI since it is one of the most active UBIs (IC_50_ = 28 nM), exhibits excellent ability to cross the membrane, and has moderate activity in a mouse model of trypanosomiasis when administered orally [14].

### Interactions in the EMP

The EMP is formed mainly by hydrophobic *Tb*MetRS residues, some of which form the original Met pocket. Insertion of R1 into the EMP prevents the binding of Met. A core of 11 residues is in contact with the R1 group in all the complexes (Figure S3A). Within the EMP, two sub-pockets can be discerned. One sub-pocket, lined mainly by Pro247, Ile248, Asn480, Tyr481 and His523, is initially filled by the sulfur atom of Met in the Met pocket, hence will be termed the ‘EMP-S’. The displacement of Val473, Trp474 and Phe522, together with Leu478, form the ‘enlarged’ part of the EMP, the sub-pocket ‘EMP-E’ (Figure 2B).

**Figure 3:**
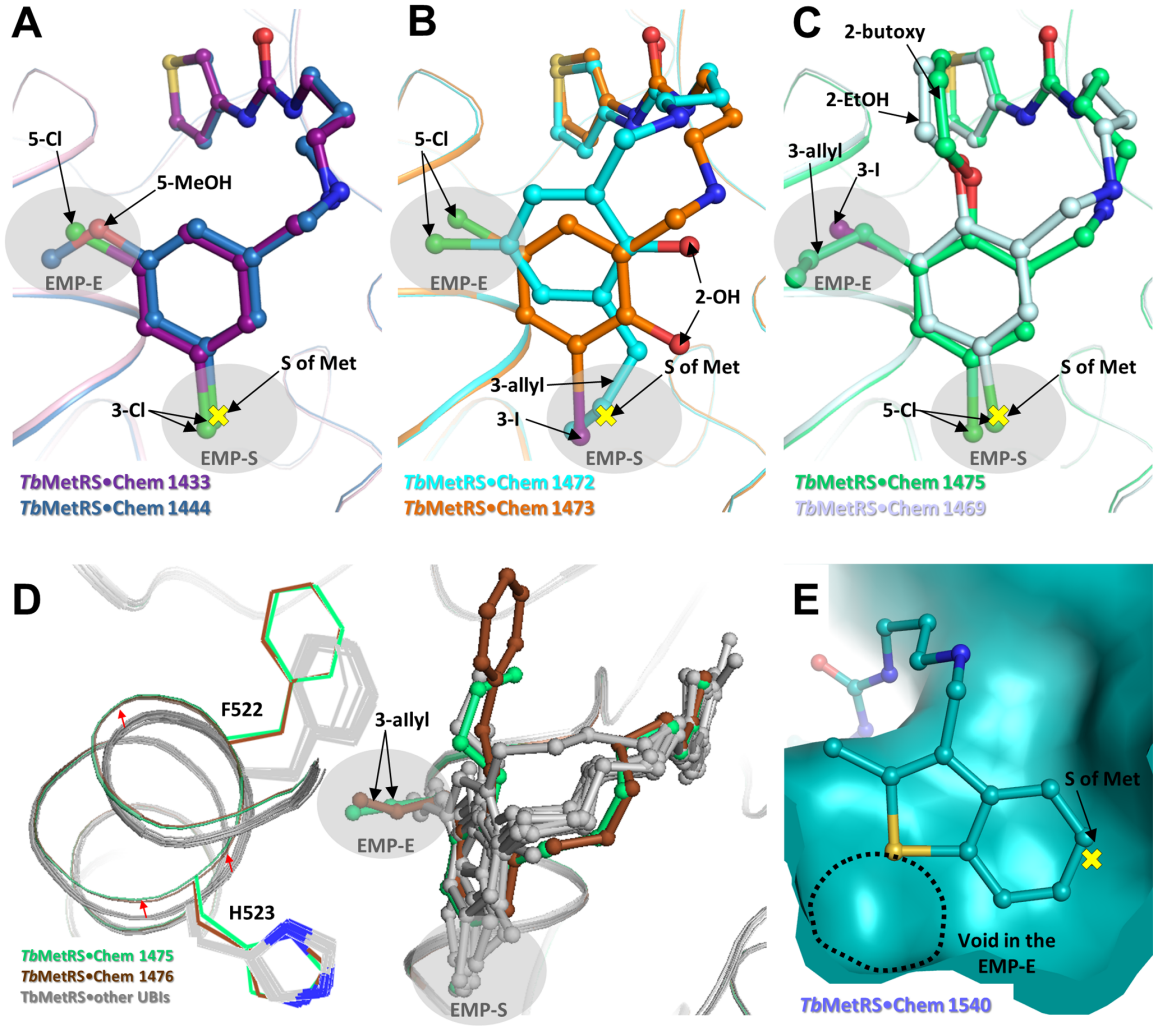
Interactions in the EMP. (A) Superpositions of Met-bound (not shown), **Chem 1433**-bound (deep purple) and **Chem 1444**-bound (blue) complexes show that a *meta*-Cl occupies the same position as the sulfur atom of Met (marked as yellow cross) in the EMP-S. The other *meta*-substituent occupies the EMP-E. *Meta*-Cl is preferred over *meta*-methoxy in the EMP-S. (B) If the *ortho*-substituent is small (hydroxyl), *meta*-allyl in **Chem 1472** (cyan) or *meta*-I in **Chem 1473** (orange) is bound to the EMP-S and *meta*-Cl is bound to the EMP-E. (C) If the *ortho*-substituent is large, *meta*-Cl is bound to the EMP-S and *meta*-butoxy in **Chem 1475** (green) or *meta*-ethoxy in **Chem 1469** (pale blue) is bound to the EMP-E. (D) Superpositions of all 15 UBI complexes indicate a consistent conformation of the EMP is selected for binding in 13 of the complexes (gray). In contrast, two other complexes, compound **Chem 1475**-bound (green) and compound **Chem 1476**-bound (brown) with large *ortho*- and *meta*-substituents in and near the EMP-E, cause minor adjustment in the conformation of the protein. Residues to the N-terminal of α-9 are displaced by an average of 0.7 Å for their C_α_ atoms as indicated by the red arrows. Displacement of Phe522 and His523 is also observed. (E) Compound **Chem 1540** (teal) is an example of inhibitors without the typical 3,5-di-halide substituted phenyl group as R1. The EMP-S is occupied but the fitting of the EMP-E is sub-optimal, resulting in a hydrophobic void and high IC_50_ value.

The more potent UBIs typically are doubly-substituted at their *meta*-positions of the phenyl group in R1, filling both sub-pockets. For example, the prototypic inhibitor **Chem 1433** is 3,5-diCl substituted. One of the *meta*-Cl atom occupies essentially the same position as the Met sulfur atom (within ∼0.6 Å), filling the EMP-S. The other *meta*-Cl atom fits into the EMP-E (Figure 3A). A few other UBIs, such as **Chem 1444** and **Chem 1415**, have the asymmetrical *meta*-substitution of 3-Cl,5-OMe groups. In both inhibitors, the 3-Cl atom is preferred over the 5-OMe in the EMP-S (Figure 3A).

Five UBIs used in this study contain a tri-substituted R1 phenyl ring. These UBIs all have a Cl atom at one *meta*-position with either an iodine or an allyl group at the other *meta*-position. In addition, one of the ortho positions is substituted with either a 2-OH, 2-EtOH, 2-butoxy or 2-benzoxy. They are less active than inhibitors with a di-*meta*-substitution but these five structures provide information about the size of substituents that can be tolerated in the two sub-pockets of the EMP. The larger *meta*-substituents of an iodine or an allyl group appear to be favored in the EMP-S compared to the EMP-E, if the *ortho*-substituent is small (2-OH in **Chem 1472** and **Chem 1473**) (Figure 3B). This is because next to the EMP-S, the space around the *ortho*-position is quite confined by the carbonyl O of Ile248 and C-β of Pro247, which are at distances of 3.7 Å and 4.5 Å, respectively, from the *ortho* carbon of R1 in the *Tb*MetRS•**Chem 1433** complex. In contrast, the space around the *ortho*-position next to the EMP-E is open. Therefore, larger *ortho* substituents do not fit into the EMP-S sub-pocket but can be accommodated by the space next to EMP-E sub-pocket (2-EtOH in **Chem 1469**, 2-butoxy in **Chem 1475** and 2-benzoxy in **Chem 1476**) (Figure 3C). This is likely to result in the insertion of large *meta* substituents in the EMP-E (3-I in **Chem 1469**, and 3-allyl in **Chem 1475** and **Chem 1476**), which is a less favorable binding pose as reflected in their high IC_50_ values (Figure 1).

Interestingly, the binding of large 2- and 3-substituents in and around the EMP-E, as seen in the complexes with **Chem 1475** and **Chem 1476**, respectively, is accompanied by conformational adjustments of the protein. Residues Asp518 to His523 in the N-terminal part of helix α-9 are displaced by an average of 0.7 Å for the C_α_ atoms, and by as much as 1.8 Å for the side chain of Phe522, to create the space needed for the insertion of the allyl group of **Chem 1475** and **Chem 1476** into the EMP-E (Figure 3D).

Structures of three inhibitors that lack a halide-substituted R1 phenyl ring were also determined. The R1 moieties of **Chem 1478**, **Chem 1509** and **Chem 1540** are 3-phenylethyne, 2,5-dichlorothiophene and 2-methyl-1-benzothiophene, respectively. While the EMP-S in these complexes is filled, the fit in the EMP-E is sub-optimal, in agreement with their weaker activities compared to the prototypic inhibitor **Chem 1433** (Figure 3E). The distances between inhibitor atoms facing the EMP-E to protein atoms forming the pocket are between 3.6 Å to 6.1 Å, creating a hydrophobic void in the pocket.

### Interactions in the AP

**Figure 4:**
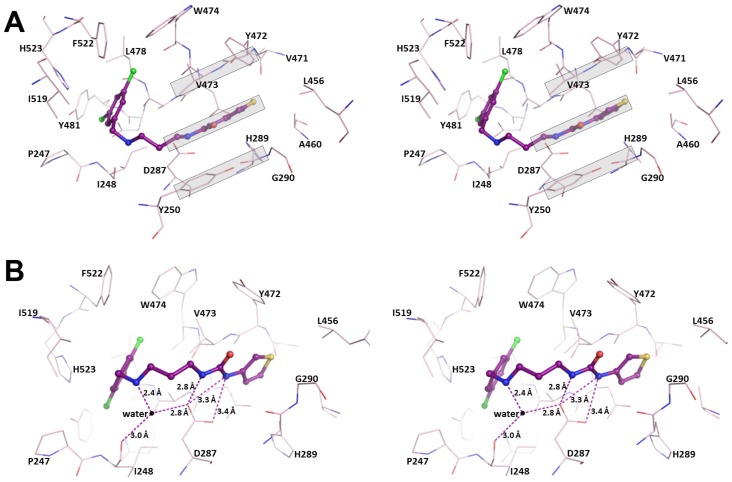
Interactions in the AP. Stereo pairs showing interaction between **Chem 1433** (ball and stick model, deep purple) and protein residues (stick model, light pink) within 4.5 Å radius of the inhibitor. (A) In the AP, the near-planar urea-R2 group is sandwiched in between the ‘walls’ formed by the similarly planar features of Tyr250 (side chain) and His289-Gly290 (peptide unit) on one side, with Val473 (side chain) and Tyr472-Val473 (peptide unit) on the other side (all boxed in gray shade). (B) The urea moiety forms crucial hydrogen bonds with Asp287, a strictly conserved residue among all MetRS. The secondary amine in the linker is bound to a water molecule (sphere, black) which in turn is hydrogen-bonded to the conserved Asp287 and the carbonyl oxygen of Ile248. Also see Figure S3B for further details of the interactions within the AP.

The AP is not present in the M-state of *Tb*MetRS but is observed in the I-state after binding to inhibitors, as a result of a large number of structural changes [13]. The AP, delineated by 10 residues, is also largely hydrophobic like the EMP (Figure S3B). There are three types of R2 moieties in the complexes determined in this study. Eleven UBIs have a 3-thiophene ring, two a phenyl ring, and two a hydroxylated phenyl ring. All the urea-R2 moieties in UBIs bind to the AP in a similar mode. The R2 group of the inhibitors is inserted between helices α-2 and α-7 of the Rossmann fold core. The near planar urea-R2 moieties slide deep into the AP mainly through stacking interactions with, on one side, the planar Tyr250 side chain and the His289-Gly290 peptide unit and, on the other side, the Tyr472-Val473 peptide unit and the Val473 side chain (Figure 4A). The urea moiety is directly connected to the R2 group and occupies the entrance to the AP. Crucially, Asp287, which originally formed hydrogen bonds with the NH of the substrate Met [13], shifts towards the inhibitor and forms new hydrogen bonds with an NH group of the urea moiety (Figure 4B). A groove connecting the EMP and the AP in the I-state is blocked in the M-state by Tyr250. A flip of the Tyr250 side chain in the I-state allows the placement of the *N*-methylpropylamine urea linker of the inhibitors.

### Flexibility in linker

The *N*-methylpropanamine linker is the most flexible part of the inhibitors. There are four rotatable bonds connecting the five atoms in this linear moiety. The flexibility of the linker is evident even after the inhibitors are bound to the protein, as densities of the linker in all complexes are almost always weaker than the other parts of the inhibitors (Figure S2). Also, linkers can adopt different conformations – in particular in compounds **Chem 1472, Chem 1475** and **Chem 1476**, the linker deviates from the most common linker conformation observed in other UBIs.

In most structures, the secondary amine group in the linker is bound to a water molecule, which is in turn H-bonded to the carboxylate of Asp287 and the carbonyl oxygen of Ile248 (Figure 4B). This water-mediated interaction with Asp287 could be particularly important in optimally positioning the Asp residue. The linker is otherwise mostly solvent exposed.

### The simultaneous binding of Chem 1433 and AMPPCP

**Figure 5:**
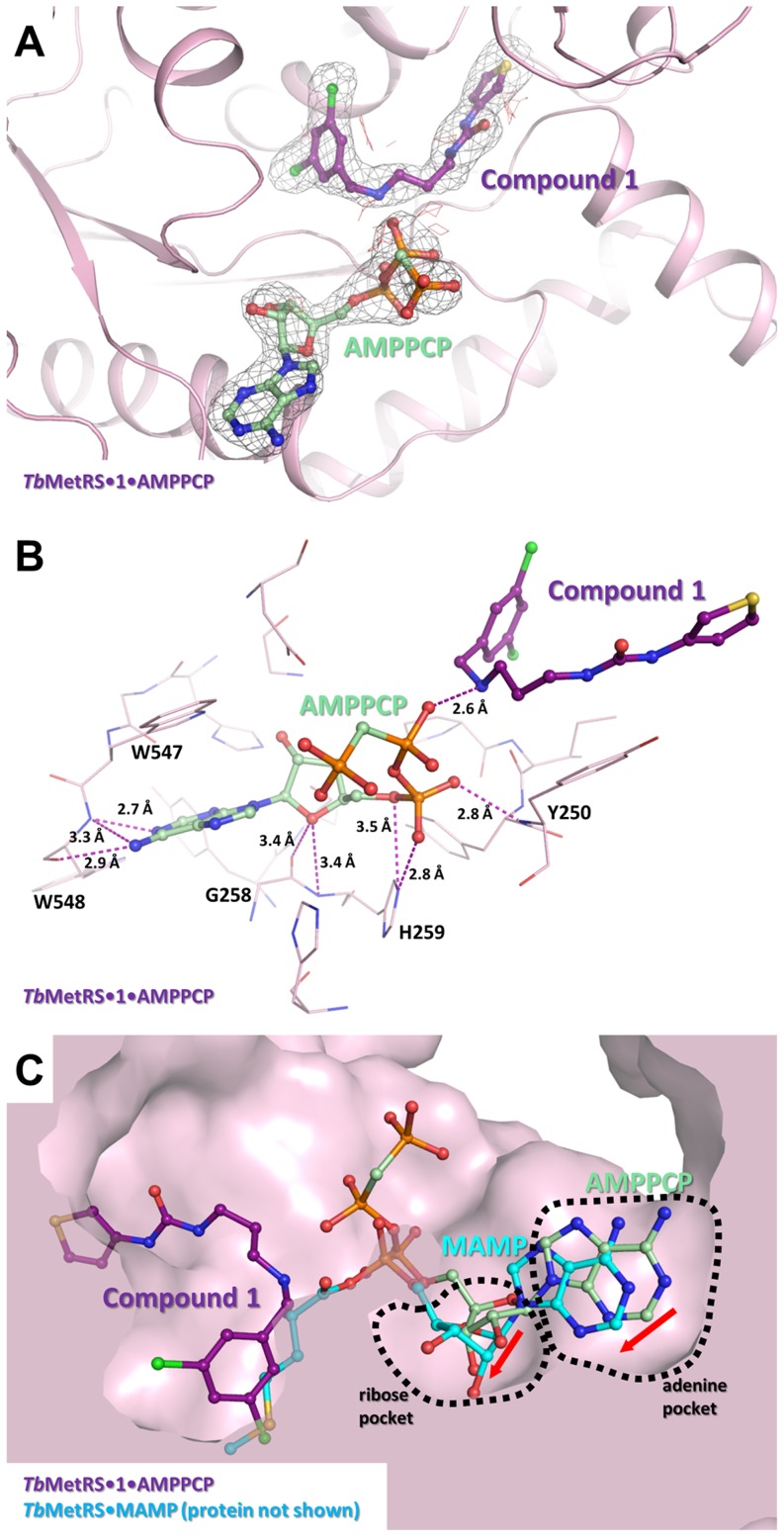
The simultaneous binding of Chem 1433 and AMPPCP. (A) The structure of *Tb*MetRS•**Chem 1433**•AMPPCP shown with the difference electron density calculated by omitting **Chem 1433** and AMPPCP, contoured at 3σ (gray is positive density, red is negative density). (B) Residues around 4.5 Å radius of AMPPCP is shown in stick model (light pink) with **Chem 1433** (deep purple) and AMPPCP (pale green) shown in ball and stick model. Possible hydrogen bonds between AMPPCP and *Tb*MetRS are shown with a dashed line. Crucially, the secondary amine in the linker of **Chem 1433** forms a strong hydrogen bond with a β-phosphate oxygen in AMPPCP (2.6 Å). (C) Superposition of *Tb*MetRS•**Chem 1433**•AMPPCP and *Tb*MetRS•MAMP (PDB: 4EG3, protein not depicted) show that the AMP moiety of MAMP (cyan) binds, on average, approximately 1.5 Å deeper into the ribose and adenine pockets (red arrow).

Superposition of the *Tb*MetRS•UBIs and *Tb*MetRS•MAMP (PDB code 4EG3 [13]) complexes revealed that the binding sites of UBIs and ATP may not overlap. Therefore, a soak was performed with **Chem 1433**, the ATP analogue AMPPCP and Mg^2+^, resulting in a structure of the ternary *Tb*MetRS•**Chem 1433**•AMPPCP complex. The binding sites for ATP in both subunits in the asymmetric unit are accessible in the crystals, demonstrated by the formation of MAMP in both active sites when ATP and Mg^2+^ are soaked into *Tb*MetRS•Met crystals [13]. Yet surprisingly, after soaking, AMPPCP does not bind to subunit A and Met remains bound essentially as without AMPPCP. In contrast, the density for AMPPCP is clear in subunit B, along with density for **Chem 1433** (Figure 5A). **Chem 1433** binding to the protein is essentially identical whether or not AMPPCP is present. Of possible significance is that one of the β-phosphate oxygen in AMPPCP is only 2.6 Å away from the amine group of the linker, with an NH…O angle of ∼135° (Figure 5B). Hence, a favorable interaction between this UBI and AMPPCP is likely to exist.

Superposition of subunit B in the current *Tb*MetRS•**Chem 1433**•AMPPCP structure and the *Tb*MetRS•MAMP structure [13] shows that the AMPPCP binding site overlaps with that of the AMP moiety of MAMP (Figure 5C). A few side chains (His256, His259 and Trp547) in the pocket are shifted slightly, by less than 2.5 Å, but the overall shape of the two pockets is rather similar to each other. However, the adenine ring and ribose sugar from MAMP bind ∼1.5 Å deeper into their respective pockets, compared to AMPPCP (Figure 5C). In the MAMP complex, stacking of the adenine ring with the Trp547 indole is also tighter than in the AMPPCP complex. Distances between atoms of the two rings range from 3.5 to 3.8 Å for MAMP in the binary *Tb*MetRS•MAMP complex, and around 4.0 to 4.5 Å for AMPPCP in the *Tb*MetRS•**1433**•AMPPCP complex.

Thermal melt experiments on *Tb*MetRS in the presence of different combinations of ligands were performed to determine the nature of the interactions between **Chem 1433** and ATP when they are bound to *Tb*MetRS. A similar analysis has been used to establish synergism between an inhibitor of human ProRS, halofuginone, and ATP [25]. An increase in T_m_ of the *Tb*MetRS•**1433** complex upon adding ATP may be an indication of synergism between **Chem 1433** and ATP in binding. Our results showed that the T_m_ of *Tb*MetRS does not change significantly in the presence of ATP-Mg^2+^ or AMPPCP-Mg^2+^. Instead, the addition of ATP-Mg^2+^ or AMPPCP-Mg^2+^ to the complex of the *Tb*MetRS•**Chem 1433** complex result in increase of T_m_ by an average of 1.6°C (n = 4, *P*<0.02) and 1.2°C (n = 4, *P*<0.05), respectively, compared to the T_m_ of *Tb*MetRS•**Chem 1433** alone (Figure S4).

## Discussion

### Interactions between *Tb*MetRS and inhibitors

The 15 new UBI structures reported here widen our understanding of key structural features of this class of inhibitors which govern their affinity for *Tb*MetRS. Some general rules based on the current complexes between *Tb*MetRS and the R1-linker-R2 type of inhibitors include:

1. a di-*meta*-substituted phenyl ring as R1 binds to the EMP in two sub-pockets;
2. a small *ortho*-substituent can be accommodated near the sub-pocket EMP-S but a large *ortho*-substituent can only be accommodated near the sub-pocket EMP-E;
3. a planar moiety as R2 binds to the AP and can be inserted between the planar ‘walls’ of the pocket;
4. the NH-C-NH moiety of the aminoquinolone or urea moieties is hydrogen-bonded to an invariable Asp in MetRS (Asp287 in *Tb*MetRS);
5. the linkers in the inhibitors are flexible and mostly solvent exposed; and
6. the UBIs are not competing with ATP for binding. Instead, a secondary amine of the linker potentially forms a H-bond with ATP.

These insights might be used to improve further the affinity of next generations of inhibitors or to maintain high affinity when modifications are introduced to improve pharmacokinetic or other properties of current compounds.

The two sub-pockets in the EMP appear to be important determinants for high affinity inhibitors. Inhibitors with the highest potency have a Cl atom that virtually coincides with the sulfur atom position in the EMP-S. Methionine is the only natural amino acid with a thiol ether side chain. It is likely that the sulfur atom is utilized as a feature for specific recognition by MetRS. Considering the similar van der Waals radii of sulfur and chlorine [26], it is tempting to suggest that a *meta*-Cl in inhibitors mimics the physico-chemical properties of sulfur in Met and therefore occupies a strikingly similar position in the EMP-S as the S of Met. In contrast, UBIs with other types of R1 group (**Chem 1478, Chem 1509** and **Chem 1540**) are unable to fill both EMP sub-pockets (Figure 3E), resulting typically in weaker activity. Large *meta*-substituents and additional *ortho*-substituents on R1 phenyl are also not well tolerated, possibly due to steric clashes (Figure 1).

We have previously proposed that ABIs bind to *Tb*MetRS through conformational selection [13]. The new complexes are consistent with this hypothesis. Despite a wide diversity of inhibitor structures being explored, including R1 groups that are either ‘oversized’ (such as **Chem 1472** and **Chem 1469**) or ‘undersized’ (such as **Chem 1478**, **Chem 1509** and **Chem 1509**), the binding pockets of all complexes are essentially identical. This supports the notion that the inhibitors ‘select’ for a pre-existing, low energy conformation in the F-state of the enzyme for binding, instead of inducing conformations of binding pockets for different inhibitors. Yet, **Chem 1475** and **Chem 1476** appear to be the exception in that the EMP is subtly changed by these inhibitors. This represents, most likely, a realistic picture of the dynamics during the inhibitor binding process where the distinction between conformational selection or induced-fit mechanism is not always sharp. An “extended conformational selection” model, in which conformational adjustment following initial conformational selection has been suggested [27]. In this case, the common conformation selected by all other inhibitors is probably not compatible with binding of **Chem 1475** and **Chem 1476** due to steric clashes. However, a subsequent minor adjustment of the EMP upon binding, albeit with a higher energy protein conformation, is possible. This unfavorable component of the free energy of binding is manifested in the higher IC_50_ values for these two inhibitors.

### Comparison between UBI and ABI in the AP

**Figure 6:**
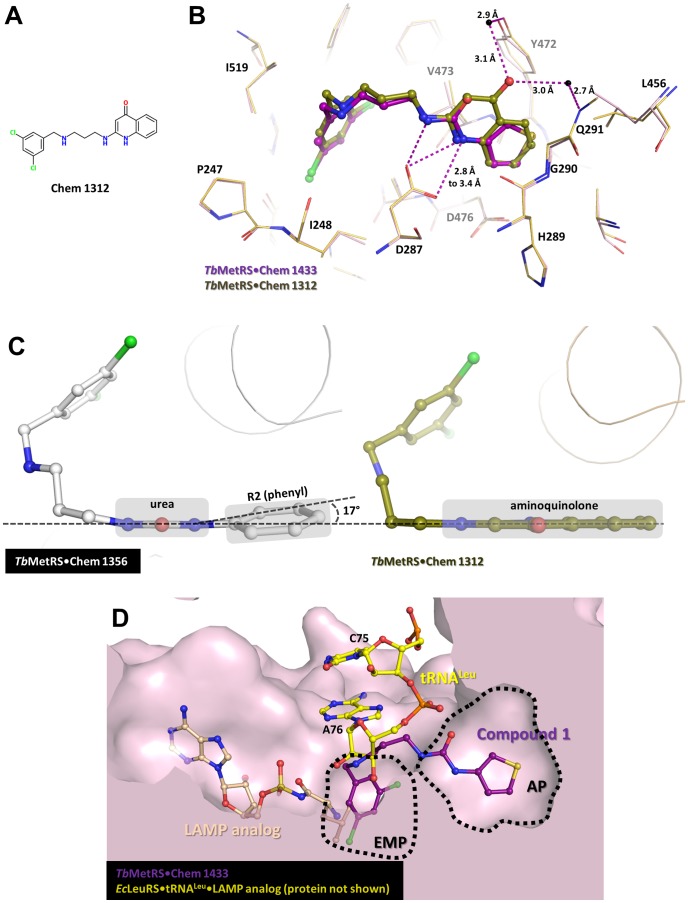
The interactions in the AP and its role in *Tb*MetRS. (A) The chemical structure of a previously reported aminoquinolone-based inhibitor (ABI) **Chem 1312** used for comparison with UBIs. (B) Superposition of *Tb*MetRS•**Chem 1433** (with **Chem 1433** being an UBI) and *Tb*MetRS•**Chem 1312** (**Chem 1312** being an ABI; PDB: 4EG5) shows that both bind to *Tb*MetRS with a similar binding mode. Contact residues within 4.5 Å of the **Chem 1433**-bound complex (light pink) and **Chem 1312**-bound complex (gold) are shown in stick model, are essentially in the same conformation. The crucial hydrogen bonds between aminoquinolone of **Chem 1312** (olive) and urea of **Chem 1433** (deep purple) with Asp287 are conserved in both complexes. One of the main differences is the presence of 4-ketone group in the aminoquinolone moiety which makes two water-mediated interactions with Gln291 and Tyr472. This water molecule is shown as black sphere. (C) The planarity of the aminoquinolone moiety in ABI is not perfectly replicated by the urea-R2 moiety in the UBIs. As an example, the interplanar angle between the urea and R2 (phenyl) in **Chem 1356** (white) is 17°, compared to the co-planar aminoquinolone ring system in **Chem 1312** (olive). (D) Superposition of *Tb*MetRS•**Chem 1433** (surface, light pink) with the ternary complex *Ec*LeuRS•tRNA^Leu^•LAMP analogue (protein not depicted; PDB: 4AQ7), shows the possible utilization of the AP by tRNA^Met^ during aminoacylation. The C75 and A76 of tRNA^Leu^ (ball and stick, yellow) occupy the entrance to the AP and either base could, via a rotation around a phosphodiester bond, be inserted into the well-conserved AP in MetRS1. **Chem 1433** (ball and stick, deep purple) and LAMP analogue (ball and stick, light brown) are shown to indicate the positions of various binding pocket and position and substrates in *Tb*MetRS.

Structurally, the key difference between ABIs and UBIs is the replacement of the aminoquinolone group in ABIs by the urea-R2 moiety in UBIs. This results in a considerable gain in membrane permeability that was reported recently although accompanied by a modest drop in activity [14]. For example, **Chem 1433**, a UBI, which corresponds to **Chem 1312**, an ABI (Figure 6A), crosses the membrane more efficiently in MDR-1 MDCKII assays but has a ∼3.5 fold less favorable IC_50_. Both aminoquinolone and urea-R2 groups bind to the AP. The hydrogen bonds between the carboxylate of Asp287 and the amines of either aminoquinolone or urea are conserved between ABIs and UBIs.

Nonetheless, two differences can be observed between UBIs and ABIs complexes. Firstly, the 4-ketone group of the aminoquinolone moiety, which is missing in the urea-R2 moiety, forms at least two water-mediated hydrogen bonds with the protein (Figure 6B). The loss of these interactions, albeit indirectly with the protein, could be part of the reason for the drop in activity. Secondly, the replacement of aminoquinolone with urea-R2 groups introduces an extra rotatable bond between the urea and the R2 groups. Given the larger number of rotatable bonds in UBIs than ABIs, and the larger flexibility of urea-R2 moiety than the conjugated system in aminoquinolone, it is reasonable to assume that UBIs have a higher conformational entropy in solution than ABIs. Therefore, upon binding, the loss of entropy of UBIs is likely higher than of ABIs, contributing to an overall less favorable free energy of binding.

The effect of the extra rotatable bond also manifests itself in a slight difference in the planarity of the AP binding moiety of inhibitors, as measured by the inter-planar angle between urea and R2 groups (Figure 1). When other parts of the inhibitors are equal, this angle is zero in ABIs with aminoquinolone, but slightly larger in UBIs with urea-thiophene, and even larger in UBIs with urea-phenyl (Figure 6C). The IC_50_ values of the inhibitors appear to be following the same trend. For example, **Chem 1312** (aminoquinolone, flat, IC_50_ = 8 nM) has a lower IC_50_ than **Chem 1433** (urea-thiophene, inter-planar angle = 9.1°, IC_50_ = 28 nM), which in turn has a lower IC_50_ than **Chem 1356** (urea-phenyl, inter-planar angle = 17°, IC_50_ = 57 nM). Another pair of comparable inhibitors, **Chem 1444** (urea-thiophene, inter-planar angle = 4.5°, IC_50_ = 19 nM) and **Chem 1415** (urea-phenyl, inter-planar angle = 16.7°, IC_50_ = 45 nM), also follows the same trend. Therefore, a planar system is probably preferred for insertion into the AP due to the stacking interactions in the pocket, although there is no clear evidence how much the small deviations from planarity may contribute to differences in affinity. It is also to be noted that the UBIs with hydroxylated-phenyl as R2 (**Chem 1387** and **Chem 1392**) appear to be deviating from the trend, possibly due to a gain of water-mediated interactions between the hydroxyl group and the protein. These differences in the planarity of the AP binding moieties may warrant more studies in the future.

### The role of the AP in MetRS

The MetRS1 subfamily, but not the MetRS2 subfamily, was previously reported to be susceptible towards a group of synthetic inhibitors similar to the ABIs [8], [9]. The AP appears to be fully accessible in the F-state of all MetRS1, but is occluded in MetRS2, mainly due to the position of the gating residue Val473 [13]. The side chain of Val473 and its flanking residues Tyr472 and Trp474 form one side of the wall for the AP. Interestingly, these residues are strictly conserved among all MetRS. Based on the now available *Tb*MetRS structures, Tyr472 and Val473 are not part of the Met or MAMP-binding pockets. The other side of the wall for the AP is formed mainly by His289 and Gly290, in which Gly290 is similarly found to be strictly conserved among all MetRS despite being more than 9 Å away from any atoms of the bound Met. Therefore the AP appears to be a well-conserved pocket, at least among all MetRS1 enzymes where Val473 does not occlude the entrance [13]. This raises the interesting questions regarding the role of the AP, located right next to the Met-binding pocket, in the normal functioning of MetRS. One option is that the pocket is involved somehow in tRNA binding, but unfortunately the available tRNA-bound structures of MetRS (PDB code 2CT8 and 2 CSX) have their tRNA acceptor arms located ∼25 Å away from the active site and provide little information about tRNA near the active site [28]. Therefore, the *Tb*MetRS•**Chem 1433** complex was superimposed onto the structure of another Class I aaRS-tRNA complex, *E. coli* LeuRS•LAMP analogue•tRNA^Leu^ (PDB code 4AQ7) [29], to determine if the AP might be involved in acceptor arm binding during aminoacylation. Although LeuRS does not possess an equivalent pocket in the position of the AP, it is interesting that the tRNA sugar moieties of both A76 and C75 are located near the entrance to the AP when superposed onto *Tb*MetRS structure (Figure 6D). Consequently, it remains possible that in MetRS1 enzymes, either A76 or C75 of tRNA^Met^ could rotate around their phosphodiester bond and place either one of its bases in the AP. Interestingly, in a recent report on the complex between human ProRS, which is a Class II aaRS, and the inhibitor halofuginone, a similar binding mode was suggested, where the inhibitor simultaneously bound to Pro- and A76-binding pockets [25].

### The simultaneous binding of inhibitors and ATP

The observation of simultaneous binding of AMPPCP and UBIs could have important implications for the development of inhibitors. The ternary *Tb*MetRS•**Chem 1433**•AMPPCP complex showed that both inhibitor and ATP can bind to *Tb*MetRS simultaneously, consequently, the inhibition is not competitive with respect to ATP (Figure 5). In addition, the favorable H-bond between AMPPCP and **Chem 1433** observed in the structure also raises the possibility of co-operativity between the two ligands. Thermal melt studies on *Tb*MetRS•**Chem 1433** in the absence and presence of ATP and AMPPCP showed an increase in melting temperature (Figure S4) in agreement with the interaction seen between AMPPCP and **Chem 1433** in the crystal structure (Figure 5). This is also supported by the kinetic data reported for similar types of inhibitor (like e.g. REP8839) in the binding of *Staphylococcus aureus* MetRS, where the inhibitor is competitive with Met but uncompetitive with ATP [9]. For example, the IC_50_ of REP8839 is 30- to 50-fold lower when a physiological concentration of ATP (2500 µM) is used, than when 25 µM of ATP is used [9]. Further, ATP is similarly reported to be synergistic regarding the binding of the inhibitor halofuginone to human ProRS [30]. Recently, a crystal structure of the ternary complex of human ProRS, halofuginone and an ATP analogue (AMPPNP), an enzyme•inhibitor•nucleotide complex similar to the *Tb*MetRS•**Chem 1433**•AMPPCP complex, was reported [25]. Potentially, the interaction of ATP with inhibitors when bound to *Tb*MetRS could be one of the considerations to be kept in mind for further optimization of these inhibitors. The high concentration of ATP present in the cell could be an advantage for the binding of this type of inhibitors. Developing inhibitors to exploit such an ATP-engaging binding mode might be also further used as a general strategy for targeting other aaRSs for inhibition.

This series of structures ascertain the binding mode of UBIs to the highly flexible target *Tb*MetRS. The structures also reveal important features of the interactions of UBIs with its target MetRS which will guide future improvement of this group of active, orally available *Tb*MetRS inhibitors. Further, the ability of UBIs to cross the BBB makes them exciting leads for obtaining drug candidates for treatment of HAT. Finally, the amino acid and possible tRNA pocket (EMP and AP)-targeting nature of these inhibitors, along with a potentially ATP-engaging binding mode, constitute a general approach to develop inhibitors of all tRNA synthetases of disease-causing pathogens, a large family of valuable drug targets.

## Supporting Information

Figure S1 **The structure of** ***Tb*** **MetRS•Chem 1433.** *Tb*MetRS•**Chem 1433** structure has two subunits in one asymmetric unit (separated by the dashed line). **Chem 1433** (ball and stick, deep purple) binds to subunit B upon soaking while Met (ball and stick) is retained in subunit A. Subunit B is colored by domain features typical of MetRS: the Rossmann-fold (green), CP domain (cyan), stem-contact fold (SCF, red), and the anticodon binding α-helix bundle (light pink). The first part of the CP domain is further divided into the CP base (purple) and CP knuckle (pale blue).
(PDF)

Figure S2 **Difference electron density of UBIs.** (A) Difference electron density map for **Chem 1433** calculated by omitting the inhibitor. Maps are contoured at 3σ level (gray is positive density, red is negative density). (B) Difference electron density map for **Chem 1356** calculated by omitting the inhibitor. Maps are contoured at 3σ level (gray is positive density, red is negative density). (C) Difference electron density map for **Chem 1387** calculated by omitting the inhibitor. Maps are contoured at 3σ level (gray is positive density, red is negative density). (D) Difference electron density map for **Chem 1392** calculated by omitting the inhibitor. Maps are contoured at 3σ level (gray is positive density, red is negative density). (E) Difference electron density map for **Chem 1444** calculated by omitting the inhibitor. Maps are contoured at 3σ level (gray is positive density, red is negative density). (F) Difference electron density map for **Chem 1415** calculated by omitting the inhibitor. Maps are contoured at 3σ level (gray is positive density, red is negative density). (G) Difference electron density map for **Chem 1472** calculated by omitting the inhibitor. Maps are contoured at 3σ level (gray is positive density, red is negative density). (H) Difference electron density map for **Chem 1473** calculated by omitting the inhibitor. Maps are contoured at 3σ level (gray is positive density, red is negative density). (I) Difference electron density map for **Chem 1475** calculated by omitting the inhibitor. Maps are contoured at 3σ level (gray is positive density, red is negative density). (J) Difference electron density map for **Chem 1476** calculated by omitting the inhibitor. Maps are contoured at 3σ level (gray is positive density, red is negative density). (K) Difference electron density map for **Chem 1469** calculated by omitting the inhibitor. Maps are contoured at 3σ level (gray is positive density, red is negative density). (L) Difference electron density map for **Chem 1478** calculated by omitting the inhibitor. Maps are contoured at 3σ level (gray is positive density, red is negative density). (M) Difference electron density map for **Chem 1509** calculated by omitting the inhibitor. Maps are contoured at 3σ level (gray is positive density, red is negative density). (N) Difference electron density map for **Chem 1540** calculated by omitting the inhibitor. Maps are contoured at 3σ level (gray is positive density, red is negative density).
(PDF)

Figure S3 **Interactions between compound Chem 1433 and** ***Tb*** **MetRS.** (A) *Tb*MetRS residues (stick model, light pink) within a 4.5 Å radius of the R1 group of **Chem 1433** in the EMP are shown. (B) *Tb*MetRS residues (stick model, light pink) within a 4.5 Å radius of the urea-R2 group of **Chem 1433** in the AP are shown.
(PDF)

Figure S4 **Thermal melting curves of** ***Tb*** **MetRS in the presence of different ligands.** *Tb*MetRS (0.1 mg/ml) is incubated in the presence of different ligands as indicated. The incubation temperature of the samples is increased from 20°C to 90°C and the fluorescence signal from the reporter dye (Sypro Orange) monitored. Melting temperatures (T_m_) are calculated from the curves as the temperature where the first derivative value is maximal. The average T_m_ value of *Tb*MetRS in the presence of **Chem 1433**, ATP and Mg^2+^ (purple dash) is 1.6°C higher than the T_m_ of *Tb*MetRS in the presence of **Chem 1433** only (n = 4, *P*<0.02). The average T_m_ of *Tb*MetRS in the presence of **Chem 1433**, AMPPCP and Mg^2+^ (black dot) is 1.2°C higher than the T_m_ of TbMetRS in the presence of **Chem 1433** only (n = 4, *P*<0.05). Changes in T_m_ of *Tb*MetRS in the presence of ATP and Mg^2+^ or AMPPCP and Mg^2+^ compared to *Tb*MetRS only are insignificant (*P*>0.3 and 0.8 respectively).
(PDF)

Table S1 **Crystallographic data collection and refinement statistics.**
(PDF)

Text S1 **Synthetic methods and procedures.**
(PDF)
