# Supplementary material for: Structures of Trypanosoma brucei Methionyl-tRNA Synthetase with Urea-Based Inhibitors Provide Guidance for Drug Design against Sleeping Sickness
Source: PLoS Negl Trop Dis. 2014 Apr 17;8(4):e2775. doi: 10.1371/journal.pntd.0002775 (PMC3990509; doi:10.1371/journal.pntd.0002775)
Supplement: Figure S1 — The structure of Tb MetRS•Chem 1433. TbMetRS•Chem 1433 structure has two subunits in one asymmetric unit (separated by the dashed line). Chem 1433 (ball and stick, deep purple) binds to subunit B upon soaking while Met (ball and stick) is retained in subunit A. Subunit B is colored by domain features typical of MetRS: the Rossmann-fold (green), CP domain (cyan), stem-contact fold (SCF, red), and the anticodon binding α-helix bundle (light pink). The first part of the CP domain is further divided into the CP base (purple) and CP knuckle (pale blue). (PDF) [file pntd.0002775.s001.pdf]

Supporting information

Structures of *Trypanosoma brucei* methionyl-tRNA synthetase with urea-based inhibitors provide guidance for drug design against sleeping sickness

Cho Yeow Koh[1], Jessica E. Kim[1], Allan B Wetzel[1], Will J. de van der Schueren[1], Sayaka Shibata[1,2], Ranae M. Ranade[3], Jiyun Liu[1], Zhongsheng Zhang[1], J. Robert Gillespie[3], Frederick S. Buckner[3], Christophe L.M.J. Verlinde[1], Erkang Fan[1] and Wim G.J. Hol[1,]*

[1]Department of Biochemistry, [2]Department of Chemistry, and [3]Department of Medicine, University of Washington, Seattle, Washington 98195, USA

*Correspondence: wghol@u.washington.edu

Figure S1.

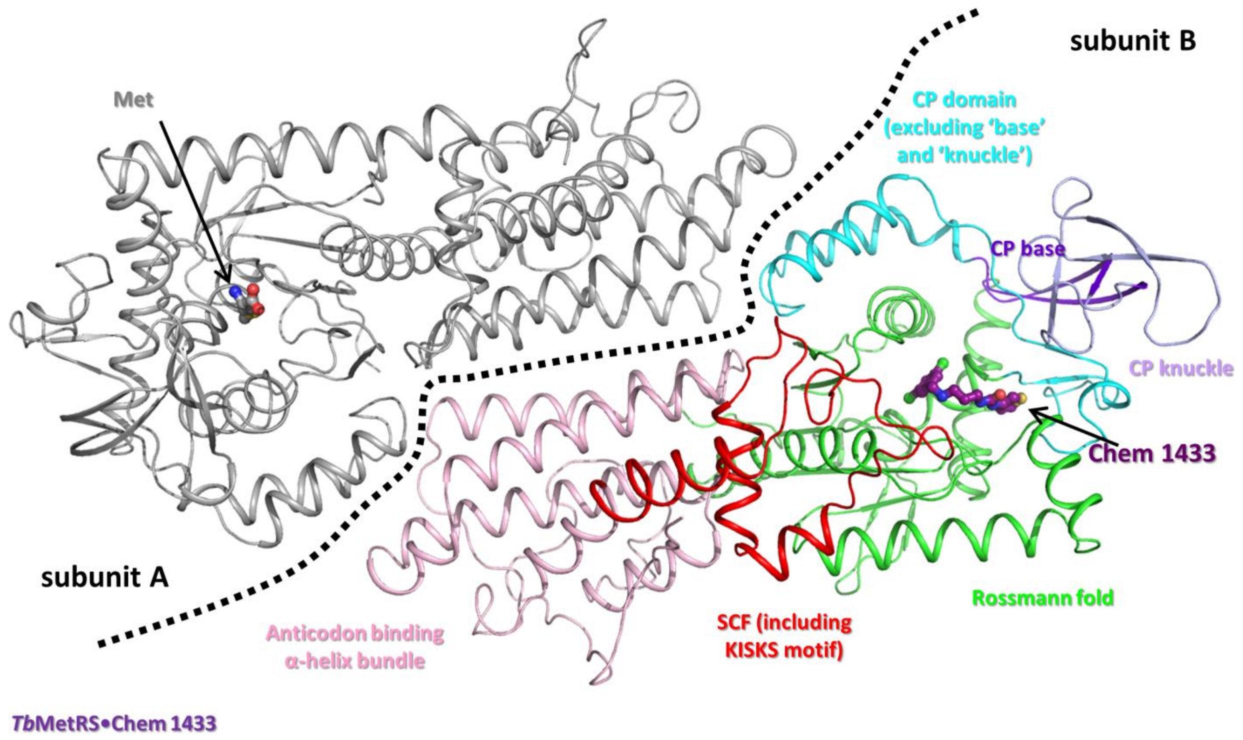

The structure of *Tb*MetRS•Chem 1433

*Tb*MetRS•**Chem 1433** structure has two subunits in one asymmetric unit (separated by the dashed line). **Chem 1433** (ball and stick, deep purple) binds to subunit B upon soaking while Met (ball and stick) is retained in subunit A. Subunit B is colored by domain features typical of MetRS: the Rossmann-fold (green), CP domain (cyan), stem-contact fold (SCF, red), and the anticodon binding α-helix bundle (light pink). The first part of the CP domain is further divided into the CP base (purple) and CP knuckle (pale blue).
