# Supplementary material for: Structures of Trypanosoma brucei Methionyl-tRNA Synthetase with Urea-Based Inhibitors Provide Guidance for Drug Design against Sleeping Sickness
Source: PLoS Negl Trop Dis. 2014 Apr 17;8(4):e2775. doi: 10.1371/journal.pntd.0002775 (PMC3990509; doi:10.1371/journal.pntd.0002775)
Supplement: Figure S2 — Difference electron density of UBIs. (A) Difference electron density map for Chem 1433 calculated by omitting the inhibitor. Maps are contoured at 3σ level (gray is positive density, red is negative density). (B) Difference electron density map for Chem 1356 calculated by omitting the inhibitor. Maps are contoured at 3σ level (gray is positive density, red is negative density). (C) Difference electron density map for Chem 1387 calculated by omitting the inhibitor. Maps are contoured at 3σ level (gray is positive density, red is negative density). (D) Difference electron density map for Chem 1392 calculated by omitting the inhibitor. Maps are contoured at 3σ level (gray is positive density, red is negative density). (E) Difference electron density map for Chem 1444 calculated by omitting the inhibitor. Maps are contoured at 3σ level (gray is positive density, red is negative density). (F) Difference electron density map for Chem 1415 calculated by omitting the inhibitor. Maps are contoured at 3σ level (gray is positive density, red is negative density). (G) Difference electron density map for Chem 1472 calculated by omitting the inhibitor. Maps are contoured at 3σ level (gray is positive density, red is negative density). (H) Difference electron density map for Chem 1473 calculated by omitting the inhibitor. Maps are contoured at 3σ level (gray is positive density, red is negative density). (I) Difference electron density map for Chem 1475 calculated by omitting the inhibitor. Maps are contoured at 3σ level (gray is positive density, red is negative density). (J) Difference electron density map for Chem 1476 calculated by omitting the inhibitor. Maps are contoured at 3σ level (gray is positive density, red is negative density). (K) Difference electron density map for Chem 1469 calculated by omitting the inhibitor. Maps are contoured at 3σ level (gray is positive density, red is negative density). (L) Difference electron density map for Chem 1478 calculated by omittin [file pntd.0002775.s002.pdf]

Supporting information

Structures of *Trypanosoma brucei* methionyl-tRNA synthetase with urea-based inhibitors provide guidance for drug design against sleeping sickness

Cho Yeow Koh[1], Jessica E. Kim[1], Allan B Wetzel[1], Will J. de van der Schueren[1], Sayaka Shibata[1,2], Ranae M. Ranade[3], Jiyun Liu[1], Zhongsheng Zhang[1], J. Robert Gillespie[3], Frederick S. Buckner[3], Christophe L.M.J. Verlinde[1], Erkang Fan[1] and Wim G.J. Hol[1,]*

[1]Department of Biochemistry, [2]Department of Chemistry, and [3]Department of Medicine, University of Washington, Seattle, Washington 98195, USA

*Correspondence: wghol@u.washington.edu

Figure S2.

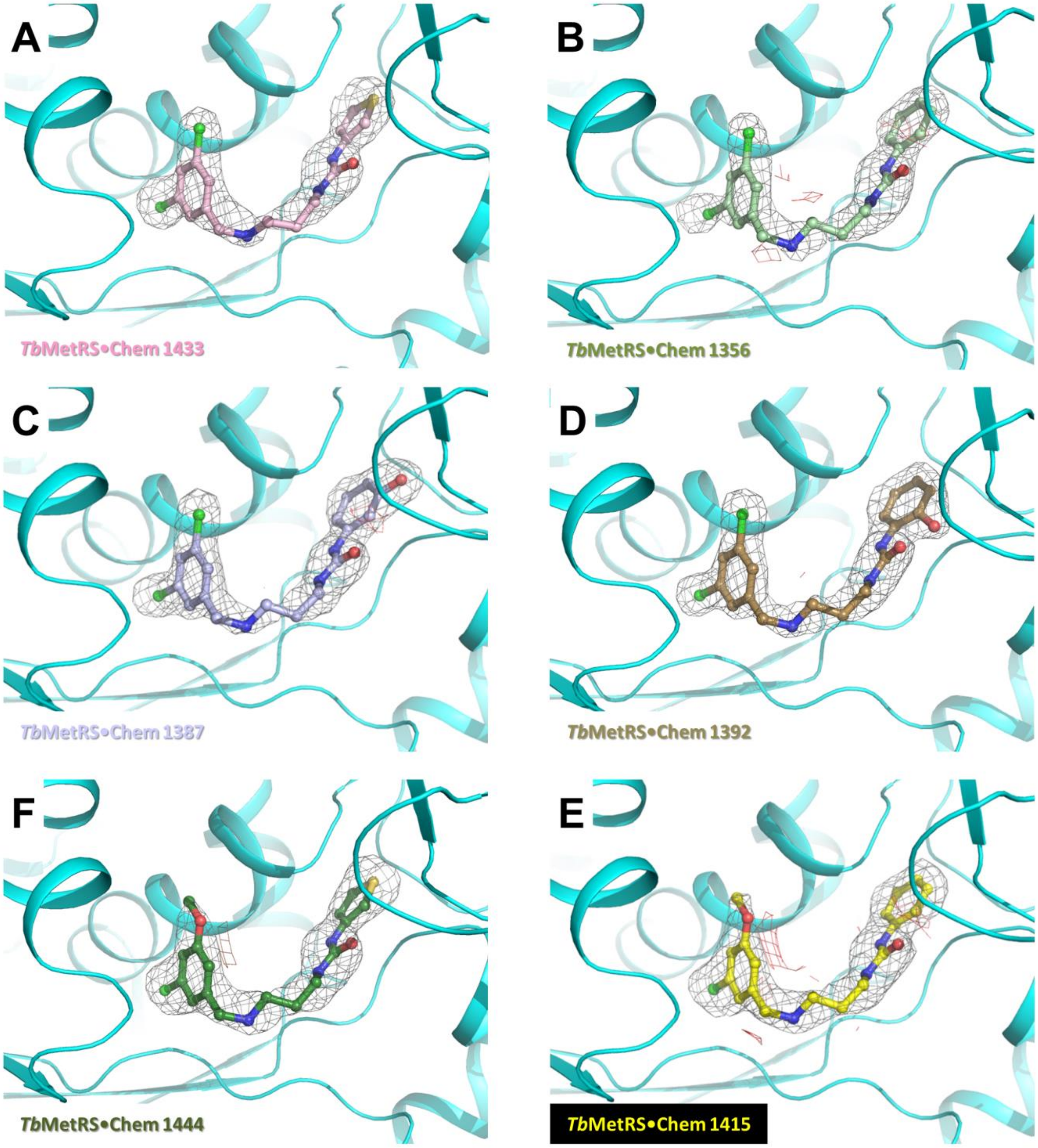
A
*Tb*MetRS•Chem 1433
B
*Tb*MetRS•Chem 1356
C
*Tb*MetRS•Chem 1387
D
*Tb*MetRS•Chem 1392
F
*Tb*MetRS•Chem 1444
E
*Tb*MetRS•Chem 1415

Figure S2 (continue).

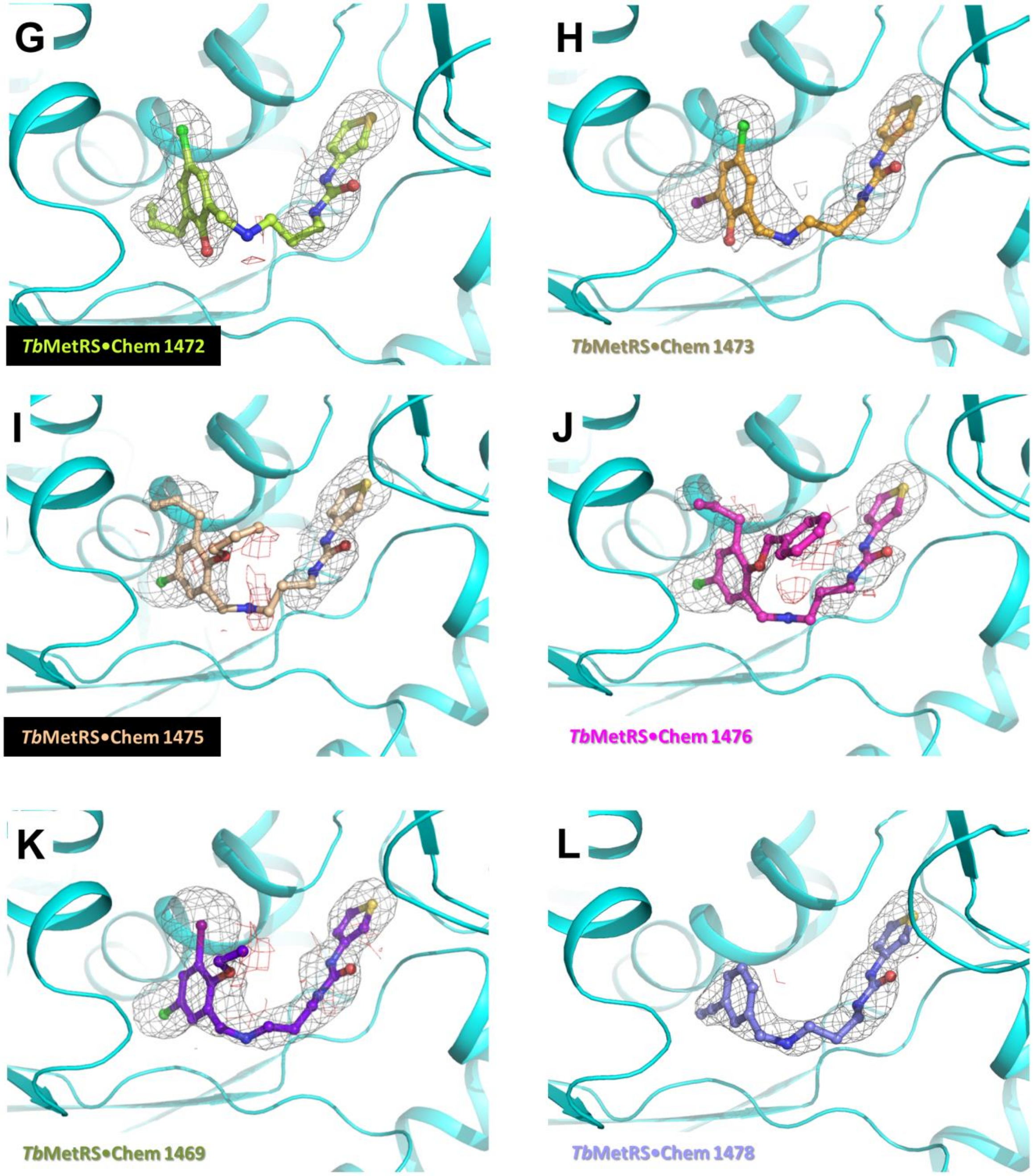

Figure S2 (continue).

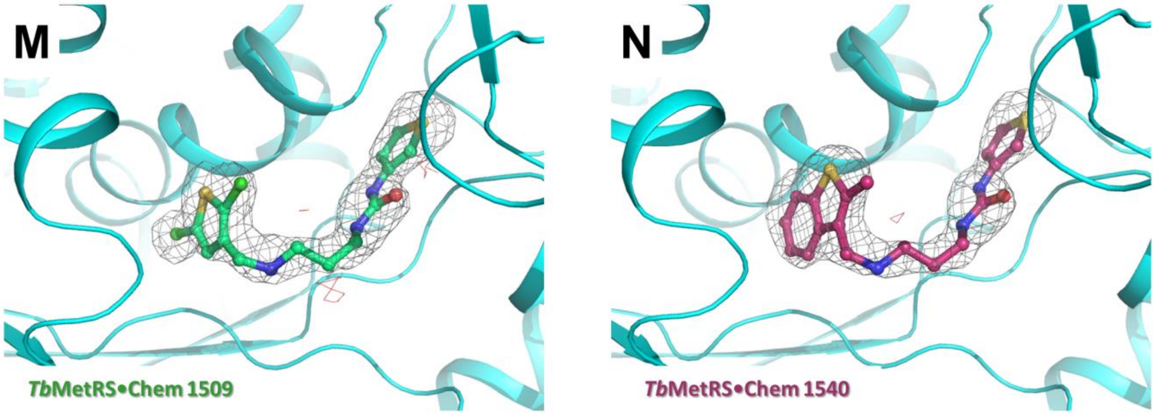

Difference electron density of UBIs.

(A) Difference electron density map for **Chem 1433** calculated by omitting the inhibitor. Maps are contoured at 3σ level (gray is positive density, red is negative density).

(B) Difference electron density map for **Chem 1356** calculated by omitting the inhibitor. Maps are contoured at 3σ level (gray is positive density, red is negative density).

(C) Difference electron density map for **Chem 1387** calculated by omitting the inhibitor. Maps are contoured at 3σ level (gray is positive density, red is negative density).

(D) Difference electron density map for **Chem 1392** calculated by omitting the inhibitor. Maps are contoured at 3σ level (gray is positive density, red is negative density).

(E) Difference electron density map for **Chem 1444** calculated by omitting the inhibitor. Maps are contoured at 3σ level (gray is positive density, red is negative density).

(F) Difference electron density map for **Chem 1415** calculated by omitting the inhibitor. Maps are contoured at 3σ level (gray is positive density, red is negative density).

(G) Difference electron density map for **Chem 1472** calculated by omitting the inhibitor. Maps are contoured at 3σ level (gray is positive density, red is negative density).

(H) Difference electron density map for **Chem 1473** calculated by omitting the inhibitor. Maps are contoured at 3σ level (gray is positive density, red is negative density).

(I) Difference electron density map for **Chem 1475** calculated by omitting the inhibitor. Maps are contoured at 3σ level (gray is positive density, red is negative density).

(J) Difference electron density map for **Chem 1476** calculated by omitting the inhibitor. Maps are contoured at 3σ level (gray is positive density, red is negative density).

(K) Difference electron density map for **Chem 1469** calculated by omitting the inhibitor. Maps are contoured at 3σ level (gray is positive density, red is negative density).

(L) Difference electron density map for **Chem 1478** calculated by omitting the inhibitor. Maps are contoured at 3σ level (gray is positive density, red is negative density).

(M) Difference electron density map for **Chem 1509** calculated by omitting the inhibitor. Maps are contoured at 3σ level (gray is positive density, red is negative density).

(N) Difference electron density map for **Chem 1540** calculated by omitting the inhibitor. Maps are contoured at 3σ level (gray is positive density, red is negative density).
