# Supplementary material for: Structures of Trypanosoma brucei Methionyl-tRNA Synthetase with Urea-Based Inhibitors Provide Guidance for Drug Design against Sleeping Sickness
Source: PLoS Negl Trop Dis. 2014 Apr 17;8(4):e2775. doi: 10.1371/journal.pntd.0002775 (PMC3990509; doi:10.1371/journal.pntd.0002775)
Supplement: Figure S3 — Interactions between compound Chem 1433 and Tb MetRS. (A) TbMetRS residues (stick model, light pink) within a 4.5 Å radius of the R1 group of Chem 1433 in the EMP are shown. (B) TbMetRS residues (stick model, light pink) within a 4.5 Å radius of the urea-R2 group of Chem 1433 in the AP are shown. (PDF) [file pntd.0002775.s003.pdf]

Supporting information

Structures of *Trypanosoma brucei* methionyl-tRNA synthetase with urea-based inhibitors provide guidance for drug design against sleeping sickness

Cho Yeow Koh[1], Jessica E. Kim[1], Allan B Wetzel[1], Will J. de van der Schueren[1], Sayaka Shibata[1,2], Ranae M. Ranade[3], Jiyun Liu[1], Zhongsheng Zhang[1], J. Robert Gillespie[3], Frederick S. Buckner[3], Christophe L.M.J. Verlinde[1], Erkang Fan[1] and Wim G.J. Hol[1,]*

[1]Department of Biochemistry, [2]Department of Chemistry, and [3]Department of Medicine, University of Washington, Seattle, Washington 98195, USA

*Correspondence: wghol@u.washington.edu

Figure S3.

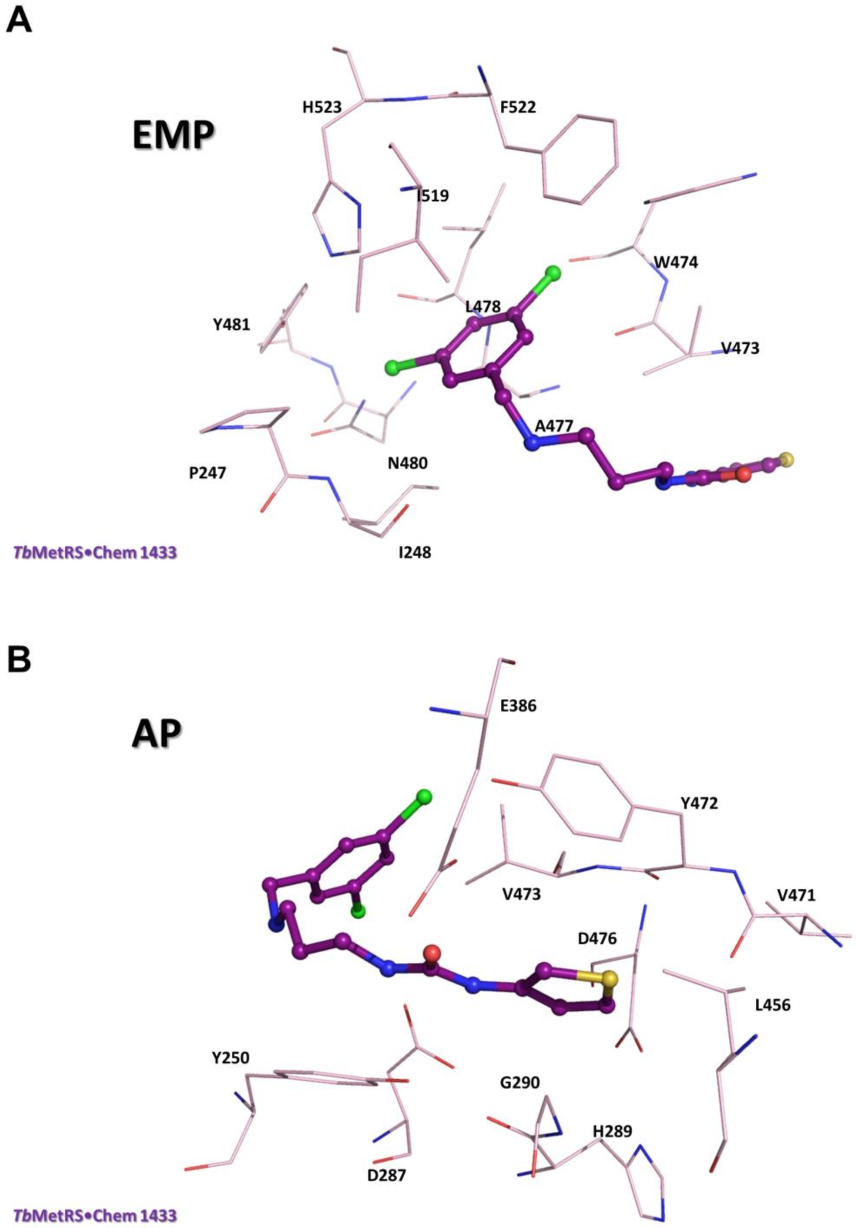

Interactions between compound Chem 1433 and *Tb*MetRS.

(A) *Tb*MetRS residues (stick model, light pink) within a 4.5 Å radius of the R1 group of **Chem 1433** in the EMP are shown.

(B) *Tb*MetRS residues (stick model, light pink) within a 4.5 Å radius of the urea-R2 group of **Chem 1433** in the AP are shown.
