# Supplementary material for: Structures of Trypanosoma brucei Methionyl-tRNA Synthetase with Urea-Based Inhibitors Provide Guidance for Drug Design against Sleeping Sickness
Source: PLoS Negl Trop Dis. 2014 Apr 17;8(4):e2775. doi: 10.1371/journal.pntd.0002775 (PMC3990509; doi:10.1371/journal.pntd.0002775)
Supplement: Figure S4 — Thermal melting curves of Tb MetRS in the presence of different ligands. TbMetRS (0.1 mg/ml) is incubated in the presence of different ligands as indicated. The incubation temperature of the samples is increased from 20°C to 90°C and the fluorescence signal from the reporter dye (Sypro Orange) monitored. Melting temperatures (Tm) are calculated from the curves as the temperature where the first derivative value is maximal. The average Tm value of TbMetRS in the presence of Chem 1433, ATP and Mg2+ (purple dash) is 1.6°C higher than the Tm of TbMetRS in the presence of Chem 1433 only (n = 4, P<0.02). The average Tm of TbMetRS in the presence of Chem 1433, AMPPCP and Mg2+ (black dot) is 1.2°C higher than the Tm of TbMetRS in the presence of Chem 1433 only (n = 4, P<0.05). Changes in Tm of TbMetRS in the presence of ATP and Mg2+ or AMPPCP and Mg2+ compared to TbMetRS only are insignificant (P>0.3 and 0.8 respectively). (PDF) [file pntd.0002775.s004.pdf]

Supporting information

Structures of *Trypanosoma brucei* methionyl-tRNA synthetase with urea-based inhibitors provide guidance for drug design against sleeping sickness

Cho Yeow Koh[1], *Jessica E. Kim*[1], *Allan B Wetzel*[1], *Will J. de van der Schueren*[1], *Sayaka Shibata*[1,2], *Ranae M. Ranade*[3], *Jiyun Liu*[1], *Zhongsheng Zhang*[1], *J. Robert Gillespie*[3], *Frederick S. Buckner*[3], *Christophe L.M.J. Verlinde*[1], *Erkang Fan*[1] *and Wim G.J. Hol*[1,*]

[1]Department of Biochemistry, [2]Department of Chemistry, and [3]Department of Medicine, University of Washington, Seattle, Washington 98195, USA

*Correspondence: wghol@u.washington.edu

Figure S4.

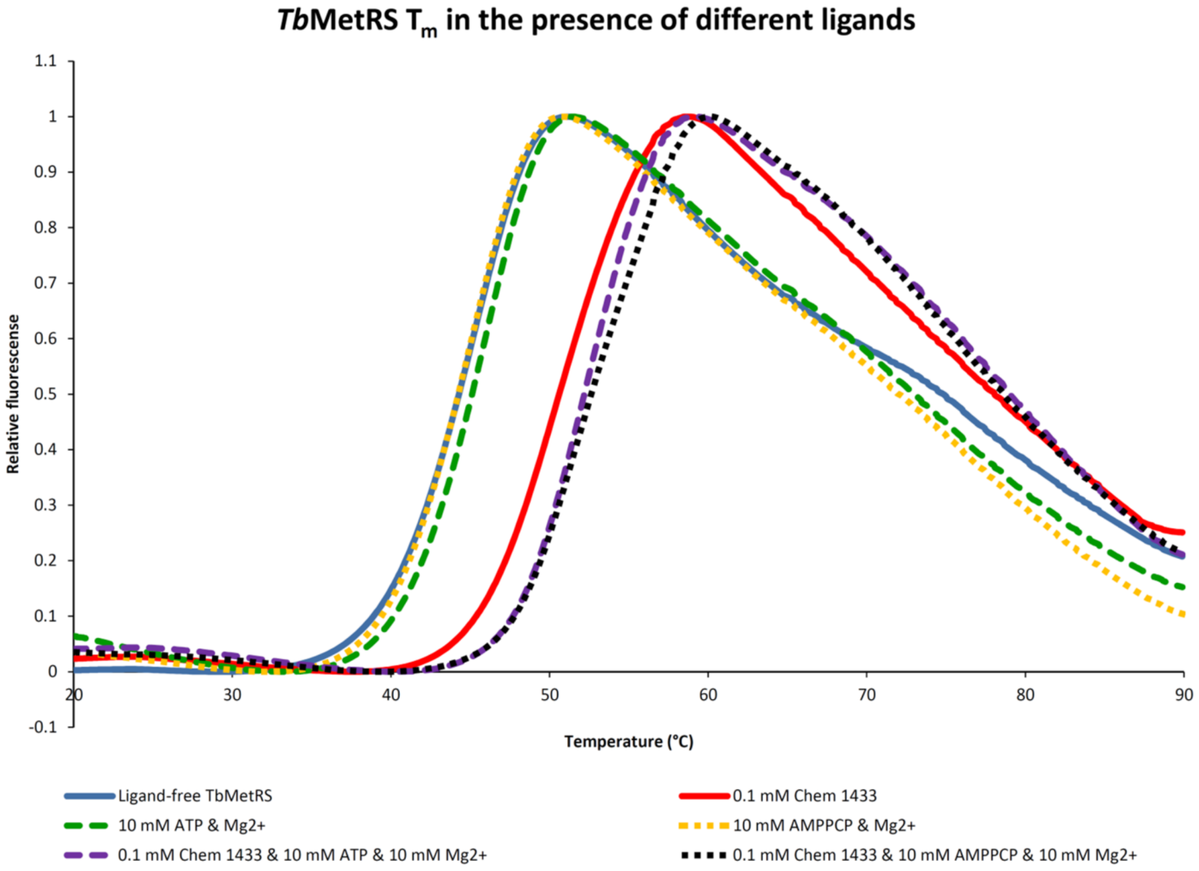

A typical thermal melting curves of *Tb*MetRS in the presence of different ligands.

*Tb*MetRS (0.1 mg/ml) is incubated in the presence of different ligands as indicated. The incubation temperature of the samples is increased from 20°C to 90°C and the fluorescence signal from the reporter dye (Sypro Orange) monitored. Melting temperatures (T_m) are calculated from the curves as the temperature where the first derivative value is maximal. The average T_m value of *Tb*MetRS in the presence of **Chem 1433**, ATP and Mg^{2+} (purple dash) is 1.6°C higher than the T_m of *Tb*MetRS in the presence of **Chem 1433** only (n = 4, $P < 0.02$). The average T_m of *Tb*MetRS in the presence of **Chem 1433**, AMPPCP and Mg^{2+} (black dot) is 1.2°C higher than the T_m of *Tb*MetRS in the presence of **Chem 1433** only (n = 4, $P < 0.05$). Changes in T_m of *Tb*MetRS in the presence of ATP and Mg^{2+} or AMPPCP and Mg^{2+} compared to *Tb*MetRS only are insignificant ($P > 0.3$ and 0.8 respectively).
