# Supplementary material for: Structures of Trypanosoma brucei Methionyl-tRNA Synthetase with Urea-Based Inhibitors Provide Guidance for Drug Design against Sleeping Sickness
Source: PLoS Negl Trop Dis. 2014 Apr 17;8(4):e2775. doi: 10.1371/journal.pntd.0002775 (PMC3990509; doi:10.1371/journal.pntd.0002775)
Supplement: Table S1 — Crystallographic data collection and refinement statistics. (PDF) [file pntd.0002775.s005.pdf]

Supporting information

Structures of *Trypanosoma brucei* methionyl-tRNA synthetase with urea-based inhibitors provide guidance for drug design against sleeping sickness

Cho Yeow Koh[1], *Jessica E. Kim*[1], *Allan B Wetzel*[1], *Will J. de van der Schueren*[1], *Sayaka Shibata*[1,2], *Ranae M. Ranade*[3], *Jiyun Liu*[1], *Zhongsheng Zhang*[1], *J. Robert Gillespie*[3], *Frederick S. Buckner*[3], *Christophe L.M.J. Verlinde*[1], *Erkang Fan*[1] *and Wim G.J. Hol*[1,*]

[1]Department of Biochemistry, [2]Department of Chemistry, and [3]Department of Medicine, University of Washington, Seattle, Washington 98195, USA

*Correspondence: wghol@u.washington.edu

TABLE S1

**Chem ID**	**1433**	**1356**	**1387**	**1392**	**1444**
**PDB ID**	4MVW	4MVX	4MVY	4MW0	4MW1
**Data collection**					
Space group	$P2_12_12_1$	$P2_12_12_1$	$P2_12_12_1$	$P2_12_12_1$	$P2_12_12_1$
Cell dimensions					
a, *b*, *c* (Å)	88.1, 105.9, 207.0	88.1, 106.0, 206.7	88.2, 106.0, 206.2	88.7, 105.9, 206.5	88.0, 105.8, 206.4
Resolution (Å)	50 – 2.90	50 – 2.55	50 – 2.30	50 – 2.20	50 – 2.50
	(3.00 – 2.90)[#]	(2.59 – 2.55)	(2.34 – 2.30)	(2.24 – 2.20)	(2.54 – 2.50)
R_{merge}	0.20 (0.83)	0.14 (0.70)	0.11 (0.79)	0.11 (0.93)	0.16 (0.85)
$I / \sigma I$	8.9 (2.1)	10.5 (2.2)	16.1 (2.1)	17.7 (2.1)	12.4 (1.9)
Completeness (%)	99.9 (100)	98.6 (99.5)	98.1 (93.4)	100 (100)	100 (100)
Redundancy	8.9 (6.4)	4.1 (4.2)	6.7 (6.6)	7.3 (7.2)	7.3 (7.4)
**Refinement**					
Resolution (Å)	30 – 2.90	30 – 2.55	30 – 2.31	30 – 2.20	30 – 2.50
No. reflections	41180	59,449	78,404	93895	64076
R_{work} / R_{free}	0.193/0.249	0.199/0.244	0.193/0.219	0.184/0.205	0.192/0.222
No. atoms					
Protein	8509	8556	8531	8541	8528
Met	9	9	9	9	9
AMPPCP	-	-	-	-	-
Inhibitor	22	23	24	24	23
Solvent ligand/ion	67	74	60	56	76
Water	268	413	560	648	427
B-factors (Å^2)					
Protein	43.4	37.2	37.0	36.5	35.6
Met	31.5	23.4	25.6	25.7	21.8
AMPPCP	-	-	-	-	-
Inhibitor	38.2	38.2	32.2	32.8	29.1
Solvent ligand/ion	58.4	64.0	61.1	62.6	59.3
Water	27.2	33.5	37.4	40.7	32.5
R.m.s. deviations					
Bond lengths (Å)	0.006	0.006	0.007	0.007	0.007
Bond angles (°)	1.06	1.08	1.18	1.15	1.17

[#]Values in parentheses are for highest-resolution shell.

Table S1. Crystallographic data collection and refinement statistics.

Chem ID	1415	1472	1473	1475	1476
**PDB ID**	4MW5	4MW2	4MW4	4MWE	4MW6
**Data collection**					
Space group	$P2_12_12_1$	$P2_12_12_1$	$P2_12_12_1$	$P2_12_12_1$	$P2_12_12_1$
Cell dimensions					
a, *b*, *c* (Å)	87.4, 105.7, 206.6	87.7, 106.1, 206.3	87.8, 106.1, 207.6	87.4, 106.1, 206.5	87.3, 106.1, 207.1
Resolution (Å)	50 – 2.35 (2.39 – 2.35)[#]	50 – 2.30 (2.34 – 2.30)	50 – 2.50 (2.54 – 2.50)	50 – 2.45 (2.49 – 2.45)	50 – 2.55 (2.59 – 2.55)
R_{merge}	0.13 (0.88)	0.11 (0.73)	0.16 (0.81)	0.14 (>1)	0.14 (0.73)
$I / \sigma I$	15.9 (2.1)	16.2 (2.1)	12.2 (2.1)	13.0 (2.0)	13.8 (2.0)
Completeness (%)	100 (99.9)	96.3 (88.7)	99.5 (98.1)	98.8 (99.8)	99.6 (97.5)
Redundancy	7.4 (7.2)	6.4 (5.9)	6.5 (6.0)	5.4 (5.2)	6.4 (5.9)
**Refinement**					
Resolution (Å)	30 – 2.35	30 – 2.30	30 – 2.50	30 – 2.45	30 – 2.56
No. reflections	76491	78393	63555	66338	59044
R_{work} / R_{free}	0.189/0.217	0.203/0.238	0.204/0.231	0.204/0.232	0.201/0.241
No. atoms					
Protein	8497	8431	8442	8467	8523
Met	9	9	9	9	9
AMPPCP	-	-	-	-	-
Inhibitor	24	25	23	28	32
Solvent ligand/ion	69	112	89	74	76
Water	544	269	298	258	338
B-factors ($Å^2$)					
Protein	36.7	47.5	40.3	49.0	45.5
Met	26.2	35.6	26.8	32.5	31.2
AMPPCP	-	-	-	-	-
Inhibitor	30.2	37.1	32.5	55.9	60.1
Solvent ligand/ion	60.3	63.0	60.4	70.3	62.3
Water	36.7	42.5	33.8	44.5	38.7
R.m.s. deviations					
Bond lengths (Å)	0.008	0.007	0.007	0.007	0.008
Bond angles (°)	1.20	1.16	1.13	1.10	1.16

[#]Values in parentheses are for highest-resolution shell.

Table S1. Crystallographic data collection and refinement statistics (continue).

**Chem ID**	**1469**	**1478**	**1509**	**1540**	**1433**•AMPPCP•Mg^{2+}
**PDB ID**	4MW7	4MW9	4MWB	4MWC	4MWD
**Data collection**					
Space group	$P2_12_12_1$	$P2_12_12_1$	$P2_12_12_1$	$P2_12_12_1$	$P2_12_12_1$
Cell dimensions					
a, *b*, *c* (Å)	87.1, 105.9, 207.3	88.0, 105.9, 207.0	88.2, 106.2, 207.0	87.7, 105.7, 206.3	88.3, 105.9, 206.7
Resolution (Å)	50 – 2.75 (2.80 – 2.75)	50 – 2.65 (2.70 – 2.65)	50 – 2.31 (2.44 – 2.31)	50 – 2.65 (2.70 – 2.65)	40 – 2.25 (2.38 – 2.25)
R_{merge}	0.14 (0.74)	0.11 (0.69)	0.16 (>1)	0.12 (0.60)	0.18 (>1)
$I / \sigma I$	10.5 (2.0)	12.3 (2.0)	12.9 (2.0)	10.0 (1.9)	11.2 (1.9)
Completeness (%)	97.4 (99.0)	100 (100)	99.9 (99.9)	99.4 (100)	99.8 (99.9)
Redundancy	4.9 (4.5)	4.0 (4.0)	8.2 (7.9)	3.9 (3.8)	7.8 (7.8)
**Refinement**					
Resolution (Å)	30 – 2.75	30 – 2.65	30 – 2.31	30 – 2.65	30 – 2.25
No. reflections	46614	53590	81144	53395	87312
R_{work} / R_{free}	0.189/0.222	0.180/0.214	0.181/0.199	0.215/0.255	0.182/0.211
No. atoms					
Protein	8347	8498	8531	8509	8523
Met	9	9	9	9	9
AMPPCP	-	-	-	-	31
Inhibitor	25	22	21	24	22
Solvent ligand/ion	91	54	74	64	76
Water	177	211	428	322	517
B-factors ($Å^2$)					
Protein	43.3	45.0	37.6	37.1	38.4
Met	27.4	29.0	24.7	23.1	28.1
AMPPCP	-	-	-	-	52.3
Inhibitor	38.4	33.4	36.1	26.5	30.1
Solvent ligand/ion	61.3	76.1	64.8	64.8	63.2
Water	32.1	34.2	36.2	36.2	38.1
R.m.s. deviations					
Bond lengths (Å)	0.007	0.007	0.007	0.007	0.007
Bond angles (°)	1.14	1.13	1.15	1.16	1.19

[#]Values in parentheses are for highest-resolution shell.

Table S1. Crystallographic data collection and refinement statistics (continue).
