# Supplementary material for: Structures of Trypanosoma brucei Methionyl-tRNA Synthetase with Urea-Based Inhibitors Provide Guidance for Drug Design against Sleeping Sickness
Source: PLoS Negl Trop Dis. 2014 Apr 17;8(4):e2775. doi: 10.1371/journal.pntd.0002775 (PMC3990509; doi:10.1371/journal.pntd.0002775)
Supplement: Text S1 — Synthetic methods and procedures. (PDF) [file pntd.0002775.s006.pdf]

Supporting information

Structures of *Trypanosoma brucei* methionyl-tRNA synthetase with urea-based inhibitors provide guidance for drug design against sleeping sickness

Cho Yeow Koh[1], *Jessica E. Kim*[1], *Allan B Wetzel*[1], *Will J. de van der Schueren*[1], *Sayaka Shibata*[1,2], *Ranae M. Ranade*[3], *Jiyun Liu*[1], *Zhongsheng Zhang*[1], *J. Robert Gillespie*[3], *Frederick S. Buckner*[3], *Christophe L.M.J. Verlinde*[1], *Erkang Fan*[1] *and Wim G.J. Hol*[1,*]

[1]Department of Biochemistry, [2]Department of Chemistry, and [3]Department of Medicine, University of Washington, Seattle, Washington 98195, USA

*Correspondence: wghol@u.washington.edu

Text S1

Synthetic Methods and Procedures

Unless otherwise stated, all chemicals were purchased from commercial suppliers and used without further purification. The final purity of all compounds was determined by analytical LCMS with Phenomenex Onyx Monolithic C18 column (4.6 mm x 100 mm). The products were detected by UV at the detection wavelength of 220 nm. All compounds were determined to be >95% pure by this method. The purification by preparative HPLC was performed on Waters Xterra Prep RP18 OBD 5μM (19 mm x 50 mm) with CH_3CN/H_2O and 0.1% TFA as eluent. The mass spectra were recorded with an Agilent Liquid Chromatograph - Ion Trap Mass Spectrometer. NMR spectra were recorded with either a Bruker 500 MHz spectrometer or a Bruker 300 MHz spectrometer at ambient temperature. Synthesis of **Chem 1433**, **Chem 1356**, **Chem 1387**, **Chem 1392**, **Chem 1444** and **Chem 1415** have been previously reported.[1] The synthesis of **Chem 1472**, **Chem 1473**, **Chem 1475**, **Chem 1476**, **Chem 1469**, **Chem 1478**, **Chem 1509** and **Chem 1540** is similar as previously reported[1] and the characterization data is presented below.

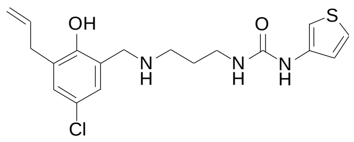

Chem 1472

Chem 1472: H^1 NMR(500 MHz, MeOD) δ 7.32-7.28 (2H, m), 7.21 (1H, d, *J* 5Hz), 7.19-7.16 (1H, m), 6.96 (1H, m), 6.05-5.95 (1H, m), 5.16-5.06 (2H, m), 5.24 (2H, s), 3.44(2H, d, *J* 10Hz), 3.34 (2H, masked by MeOD), 3.14 (2H, t, *J* 10Hz), 2.05-1.92 (2H, m). MS (ESI) $(M + H)^+$= 380.7.

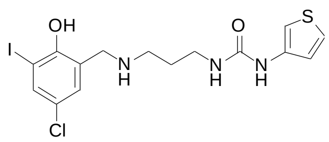

Chem 1473

Chem 1473: H^1 NMR(500 MHz, MeOD) δ 7.84 (1H, d, *J* 10Hz), 7.48 (1H, d, *J* 2Hz), 7.31 (1H, m), 7.19 (1H, m), 6.97 (1H, m), 4.28 (2H, s), 3.34(2H, t, *J* 5Hz), 3.15 (2H, t, *J* 10Hz), 1.91-1.98 (2H, m). MS (ESI) $(M+H)^+$=466.6.

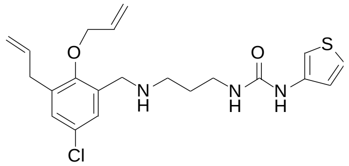

Chem 1475

Chem 1475: H^1 NMR(500 MHz, MeOD) δ 7. 47 (1H, d, *J* 2Hz), 7.36 (1H, d, *J* 2Hz), 7.31 (1H, *J* 5Hz), 7.18 (1H, d, *J* 5Hz), 6.98 (1H, d, *J* 5Hz), 6.12-6.20 (1H, m), 5.94-6.04 (1H, m), 5.47 (1H, d, *J* 15Hz), 5.31 (1H, d, *J* 10Hz), 5.15 (2H, t, *J* 10Hz), 4.45 (2H, d, *J* 2Hz), 4.24 (2H, s), 3.38-3.39 (4H, m), 3.14 (2H, t, *J* 5Hz), 2.05-1.90 (2H, m). MS (ESI) $(M+H)^+$=420.7.

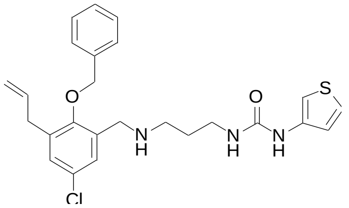

Chem 1476

Chem 1476: H^1 NMR(500 MHz, MeOD) δ 7.40-7.40 (7H ,m), 7.32 (1H, m), 7.19 (1H ,m), 6.98 (1H, m), 6.04-5.97 (1H ,m), 5.12-5.22 (2H, m), 4.99 (2H, s), 4.12 (2H, s), 3.29 (2H, t, *J* 5Hz), 2.99 (2H, t, *J* 5Hz), 1.86-1.81 (2H, m). MS (ESI) $(M + H)^+$=470.7.

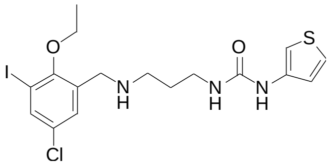

Chem 1469

Chem 1469: H^1 NMR(300 MHz, $CDCl_3$) δ 7.74 (1H, d, *J* 2.5HZ), 7.44 (1H, d, *J* 2.5), 7.10-7.16 (2H, m), 6.92 (1H, m), 3.93 (4H, m), 3.14-3.33 (2H, m), 2.85 (2H, t, *J* 6.1Hz), 1.74 (2H, m), 1.42 (3H, t, *J* 7.0Hz). MS (ESI) $(M + H)^+$= 494.7.

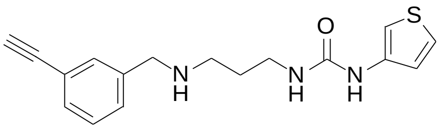

Chem 1478

Chem 1478: H^1 NMR(300 MHz, $CDCl_3$) δ 7.53 (1H, s), 7.43-7.49 (1H, m), 7.36-7.42 (1H, m), 7.28 (1H, t, *J* 7.7Hz), 7.14 (1H, m), 7.09 (1H, m), 6.87 (1H, dd, *J* 3.2Hz, 1.4Hz), 3.94 (2H, s), 3.24 (2H ,m), 3.09 (1H, s), 2.94 (2H, m), 1.74 (2H, m). MS (ESI) $(M +H)^+$= 314.6.

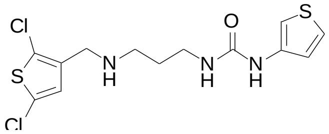

Chem 1509

Chem 1509: H^1 NMR(500 MHz, $CDCl_3$) δ 7.28 (1H, t, *J* 5Hz), 7.18 (1H, t, *J* 5Hz), 7.04 (1H, s), 6.98 (1H, dd, *J* 10Hz, 5Hz), 4.02 (2H, s), 3.43 (2H, t, *J* 5Hz), 3.06 (2H, t, *J* 5Hz), 2.04-2.01 (2H, m). MS (ESI). $(M +H)^+$=365.3.

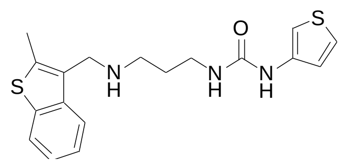

Chem 1540

Chem 1540: H^1 NMR(500 MHz, $CDCl_3$) δ 7.93 (1H ,d, *J* 5Hz), 7.45 (1H, t, *J* 10Hz), 7.38 (1H, t, *J* 5Hz), 7.271-7.31 (2H, m), 7.03 (1H, d, *J* 5Hz), 6.94 (1H, d, *J* 5Hz), 4.45 (2H, s), 3.36 (2H, t, *J* 5Hz), 3.26 (2H, t, *J* 10Hz), 2.70 (3H, s), 2.00-1.94 (2H, m). MS (ESI) $(M + H)^+$=360.5.

REFERENCES

1. Shibata S, Gillespie JR, Ranade RM, Koh CY, Kim JE, et al. (2012) Urea-based inhibitors of Trypanosoma brucei methionyl-tRNA synthetase: selectivity and in vivo characterization. J Med Chem 55: 6342-6351.
